# Mapping architectural and transcriptional alterations in non-lesional and lesional epidermis in vitiligo

**DOI:** 10.1038/s41598-017-10253-w

**Published:** 2017-08-29

**Authors:** Archana Singh, Vishvabandhu Gotherwal, Päivi Junni, Vinaya Vijayan, Manisha Tiwari, Parul Ganju, Avinash Kumar, Pankaj Sharma, Tanveer Fatima, Aayush Gupta, Ananthaprasad Holla, Hemanta K. Kar, Sangeeta Khanna, Lipi Thukral, Garima Malik, Krishnamurthy Natarajan, Chetan J. Gadgil, Riitta Lahesmaa, Vivek T. Natarajan, Rajni Rani, Rajesh S. Gokhale

**Affiliations:** 1 CSIR-Institute of Genomics and Integrative Biology, Mathura Road, New Delhi, India; 2 National Institute of Immunology, ArunaAsaf Ali Marg, New Delhi, India; 3 Academy of Scientific and Innovative Research, New Delhi, India; 4 Turku Centre for Biotechnology, University of Turku and ÅboAkademi University, Turku, Finland; 5 CSIR-National Chemical Laboratory, Chemical Engineering Division, Pune, India; 6 Department of Dermatology, Post Graduate Institute for Medical Education and Research (PGIMER), Dr. Ram Manohar Lohia Hospital, New Delhi, India; 7 Department of Dermatology, Dr. D. Y. Patil Medical College, Pimpri, Pune, India; 8 MelanoSite, Center for Advanced Vitiligo Treatment and Collaborative Pigment Cell Research, New Delhi, India; 9 School of Life Sciences, Jawaharlal Nehru University, New Delhi, India; 10 Jawaharlal Nehru Centre for Advanced Scientific Research, Bengaluru, India

## Abstract

In vitiligo, chronic loss of melanocytes and consequent absence of melanin from the epidermis presents a challenge for long-term tissue maintenance. The stable vitiligo patches are known to attain an irreversible depigmented state. However, the molecular and cellular processes resulting in this remodeled tissue homeostasis is unclear. To investigate the complex interplay of inductive signals and cell intrinsic factors that support the new acquired state, we compared the matched lesional and non-lesional epidermis obtained from stable non-segmental vitiligo subjects. Hierarchical clustering of genome-wide expression of transcripts surprisingly segregated lesional and non-lesional samples in two distinct clades, despite the apparent heterogeneity in the lesions of different vitiligo subjects. Pathway enrichment showed the expected downregulation of melanogenic pathway and a significant downregulation of cornification and keratinocyte differentiation processes. These perturbations could indeed be recapitulated in the lesional epidermal tissue, including blunting of rete-ridges, thickening of stratum corneum and increase in the size of corneocytes. In addition, we identify marked increase in the putrescine levels due to the elevated expression of spermine/spermidine acetyl transferase. Our study provides insights into the intrinsic self-renewing ability of damaged lesional tissue to restore epidermal functionality in vitiligo.

## Introduction

Vitiligo is a multifactorial complex skin disorder characterized by patchy depigmented epidermal skin. The lesional skin from vitiligo subjects is characterized by the conspicuous absence of the pigment producing cells, melanocytes. Within these cells, melanin pigment is synthesized and deposited in specialized organelles called melanosomes, which are then transferred to the neighboring keratinocytes, constituting the minimal functional epidermal melanin unit in the human skin^1^. The presence of these pigment granules prevents ultraviolet radiation-mediated DNA damage and protects the skin from cutaneous malignancies^2, 3^. Due to the selective loss of melanocytes during vitiligo, keratinocytes in these regions lack pigment and are vulnerable to environmental insults. Surprisingly contrary to this logical reasoning, vitiligo condition is increasingly considered to provide protection from melanoma and non-melanoma skin cancers^4, 5^.

Due to the apparent disappearance of melanocytes, the primary clinical management for vitiligo has focused on repopulating melanocyte cells as well as suppressing the immune response^6^. However, some of the recent studies propose a possible role of altered keratinocytes resulting in loss of homeostasis that may be contributing to the aetiopathogenesis of this disorder^7^. In addition to the conspicuous loss of melanocytes, reportedly the lesional skin also exhibits epidermal vacuolization, thickening of basement membrane, presence of T cell infiltrates and degenerating keratinocytes^8, 9^. Interestingly, ultrastructural studies on the non-lesional skin of vitiligo subjects also report altered keratinocytes, including presence of extracellular granular material and vacuolar changes in basal keratinocytes, epidermal and dermal lymphocyte infiltrates and melanophages in the upper dermis^10, 11^. Further, vitiligo patients are reported to exhibit systemic as well as localized oxidative stress^12, 13^, and millimolar levels of H_2_O_2_ are reported to be present in their epidermis^14^. This has prompted researchers to propose that even the apparently normal skin of patient may be contributing towards the disease pathogenesis and vitiligo therefore is treated as a systemic disorder^15^. Paradoxically, several studies report successful repigmentation of lesional skin using autologous transplantations of epidermal cells derived from gluteal non-lesional sites^16, 17^, suggesting that the non-lesional skin is ‘competent’ and is capable of restoring pigmentation of the transplanted lesional sites.

Previous genome-wide expression studies on intact biopsies from lesional and non-lesional skin compared to healthy controls indicated a functional role of natural killer cells and autoimmunity in melanocyte loss^18^. In another study, comparison of transcriptome between melanocyte cultures from the asymptomatic non-lesional vitiligo skin with healthy donors showed differential expression of genes involved in the regulation of melanosome maturation and antigen presentation, providing valuable insights into disease pathomechanisms^19^. Independent studies have suggested that lesional keratinocytes are prone to apoptosis and are unable to secrete melanocyte-sustaining factors such as the stem cell factor (SCF)^20, 21^. While each of these studies highlights one component of disease manifestation, together these observations suggest the lesional vitiligo skin is likely to have several perturbations.

To comprehend interplay of inductive signals and cell intrinsic factors involved in vitiligo, we compared the matched lesional and non-lesional epidermis. Incidentally, skin not only shows extensive heterogeneity across individuals in a given population, but differences are also reported across various anatomical sites within an individual^22^. We therefore designed our study to first perform the pairwise comparison of the lesional and non-lesional skin of the same individual and then compare the data across various subjects. The lesional skin samples (L) were procured from different anatomical sites of vitiligo patients, while the non-lesional (NL) skin biopsies were obtained from hip/upper thigh. In this study, we have performed comparison between the lesional (L) and gluteal non-lesional (NL) epidermis at architectural, cellular and molecular levels to identify vitiligo-specific signatures. Pair-wise analysis reduced the heterogeneity, which enabled us to comprehensively delineate vitiligo-specific perturbations in the skin, as a consequence of the melanocyte loss.

## Results

### Transcriptome profiling reveals pervasive changes in lesional epidermis in vitiligo

**Figure 1:**
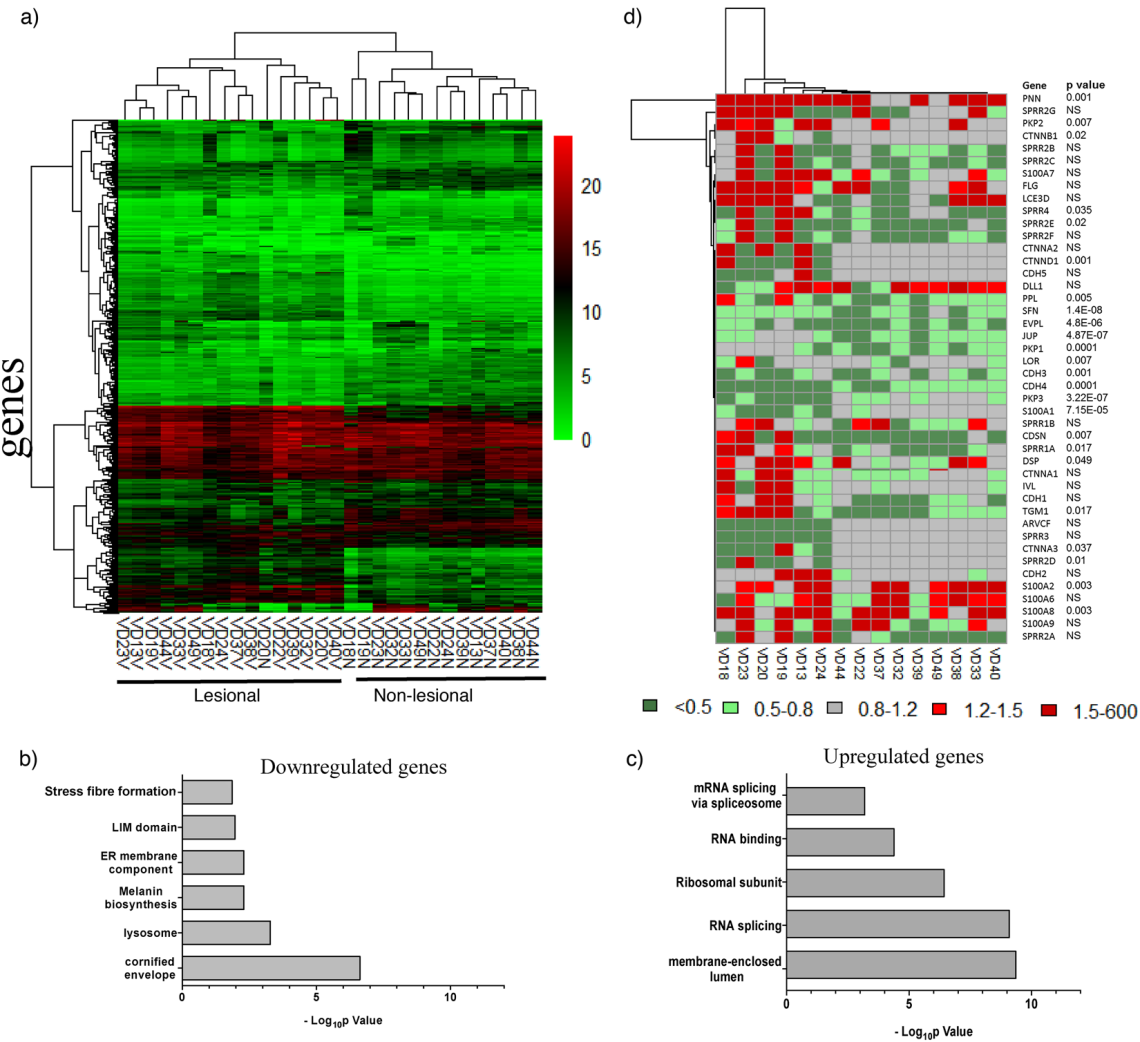
Dominant signature of keratinocyte pathology in the lesional vitiligo skin. (**a**) Clustering of samples based on average normalized expression values from microarray segregates NL and L skin (n = 15). Enrichment analysis of top 1% of (**b**) down regulated and (**c**) up regulated genes was performed by DAVID bioinformatics resource. Negative log transformed p-value of the enrichment of the pathways was calculated and represented as a bar graph. (**d**) Transcriptional regulation of genes involved in maintaining epidermal integrity and cell-cell adhesion in keratinocytes. Color scheme-Red- upregulated; green- downregulated; grey- not regulated in lesional as compared to matched non-lesional skin.

To understand molecular changes in vitiligo, we performed genome-wide transcriptome analysis from matched lesional and non-lesional epidermis derived from punch biopsies obtained from 15 vitiligo subjects (details in Table 1). Total RNA was isolated and microarray hybridization carried out using human whole genome Illumina WG-6 microarray platform. Hierarchical clustering of the average normalized expression values for all the genes, to our surprise, clustered the lesional and non-lesional samples in discrete clades. This suggested a concordant enrichment of vitiligo-specific transcriptome signatures in the lesional skin (Fig. 1a). Paired t-test analysis (p < 1 × 10^−5^) yielded a total of 1705 genes including 786 upregulated and 919 downregulated genes that showed consistent differential regulation in all 15 subjects indicating a concerted footprint of the transcriptome in vitiligo epidermis.

**Table 1:** Details of samples included in histology & transcriptome studies.

S. No.	Patient ID	Age (years)	Sex	Type of Vitiligo	Age at onset (years)	Site of non-lesional punch	Site of lesional punch
**Details of samples included in histology studies**							
1.	VD107	59	M	Vulgaris	33	Gluteal	Lower leg
2.	VD108	24	M	Vulgaris	21	Gluteal	Wrist
3.	VD131	46	F	Vulgaris	45	Gluteal	Thigh
4.	VD135	23	F	Vulgaris	5	Gluteal	Upper back
5.	VD144	24	M	Vulgaris	18	Gluteal	Thigh
6.	VD155	16	M	Vulgaris	7	Gluteal	Lower leg
7.	DYP005	45	F	Vulgaris	44	Lateral thigh	Thigh
8.	RML25	40	F	Vulgaris	38	Gluteal	Face
9.	DYP003	27	F	Vulgaris	16	Lateral thigh	Thigh
10.	DYP004	23	M	Vulgaris	16	Lateral thigh	Upper back
11.	DYP007	32	F	Vulgaris	28	Lateral thigh	Lower leg
12.	VD128	18	M	Vulgaris	11	Gluteal	Thigh
13.	RML002	24	M	Vulgaris	23	Gluteal	Hand
14.	RML018	23	M	Vulgaris	22	Gluteal	Lower leg
15.	RML03	25	F	Vulgaris	24	Gluteal	Groin
**Details of samples included in genome-wide transcriptome studies**							
1.	VD39	28	F	Vulgaris	14	Gluteal	Near knee
2.	VD22	20	F	Vulgaris	9	Gluteal	Lower leg
3.	VD20	20	F	Vulgaris	7	Gluteal	Lower leg
4.	VD32	26	F	Vulgaris	14	Gluteal	Lower leg
5.	VD37	25	F	Vulgaris	11	Gluteal	Wrist
6.	VD40	20	M	Vulgaris	09	Gluteal	Lower leg
7.	VD49	10	F	Vulgaris	NA	Gluteal	Forearm
8.	VD23	13	F	Vulgaris	03	Gluteal	Forehead
9.	VD33	26	M	Vulgaris	NA	Gluteal	Lower leg
10.	VD24	45	F	Vulgaris	36	Gluteal	Neck
11.	VD38	16	F	Vulgaris	06	Gluteal	Forearm
12.	VD44	24	F	Vulgaris	12	Gluteal	Lower leg
13.	VD13	21	M	Vulgaris	18	Gluteal	Lower leg
14.	VD19	21	M	Vulgaris	NA	Gluteal	Forearm
15.	VD18	NA	F	Vulgaris	NA	Gluteal	Lower leg

Gene Set Enrichment Analysis using DAVID suite identified several distinct biological processes that were altered among the upregulated and downregulated genes (Fig. 1b,c). The biological processes that were upregulated included ribosome biogenesis and RNA processing, while cornification and melanin biosynthesis were amongst the major downregulated processes (Fig. 1b,c and Supplementary Table 1). Whereas the apparent down regulation of melanin biosynthetic pathway is consistent with the loss of melanocyte population from the lesional epidermis, alterations in the keratinocyte-specific functions such as cornification was unanticipated. Transcriptomic analyses thus suggest biological processes that were previously not connected to vitiligo.

### Expression profile of genes involved in keratinocyte differentiation and cornification is altered in lesional epidermis

Microarray analyses of epidermal tissue suggested dysregulation of keratinocyte-specific processes in the lesional vitiligo skin. We therefore examined the keratinocyte-specific pathways in detail to understand implications of these gene expression changes. Keratinocytes undergo programmed differentiation to form terminally differentiated corneocytes, a process that is intricately linked to epidermal differentiation and cell-cell adhesion. Keratinocytes also express different adhesion proteins in various layers of the stratified epidermis. Indeed, the expression of genes involved in keratinization and epidermal differentiation were significantly altered in the lesional skin (Fig. 1d). While stratifin (SFN), corneodesmosin (CDSN), envoplakin (EVPL), transglutaminase-1 (TGM-1) and periplakin (PPL) mRNAs were significantly downregulated in the lesional epidermis, the expression of filaggrin (FLG) and involucrin (IVL) were not significantly altered (Fig. 1d). Desmosome and adherens junction components such as junctional protein (JUP) and plakophillins PKP1 and PKP3 were downregulated while PKP2 was upregulated in lesional samples. Desmoplakin (DSP), a component of desmosome complex, did not show a clear-cut pattern of regulation. The differential transcriptional regulation of adhesion components in the lesional epidermis suggested a possible aberration in the adhesion properties of keratinocytes in vitiligo (Fig. 1d). Two-way clustering of the genes involved in maintaining differentiation and adhesion is shown in Supplementary Fig. S1. Validation of some of the important genes involved in epidermal differentiation including TGM1 and CDSN, by real time PCR confirmed consistent down regulation in most of the lesional samples (Supplementary Fig. S2). These data suggest substantial changes in the lesional keratinocytes.

### Tissue architecture is perturbed in lesional vitiligo epidermis

**Figure 2:**
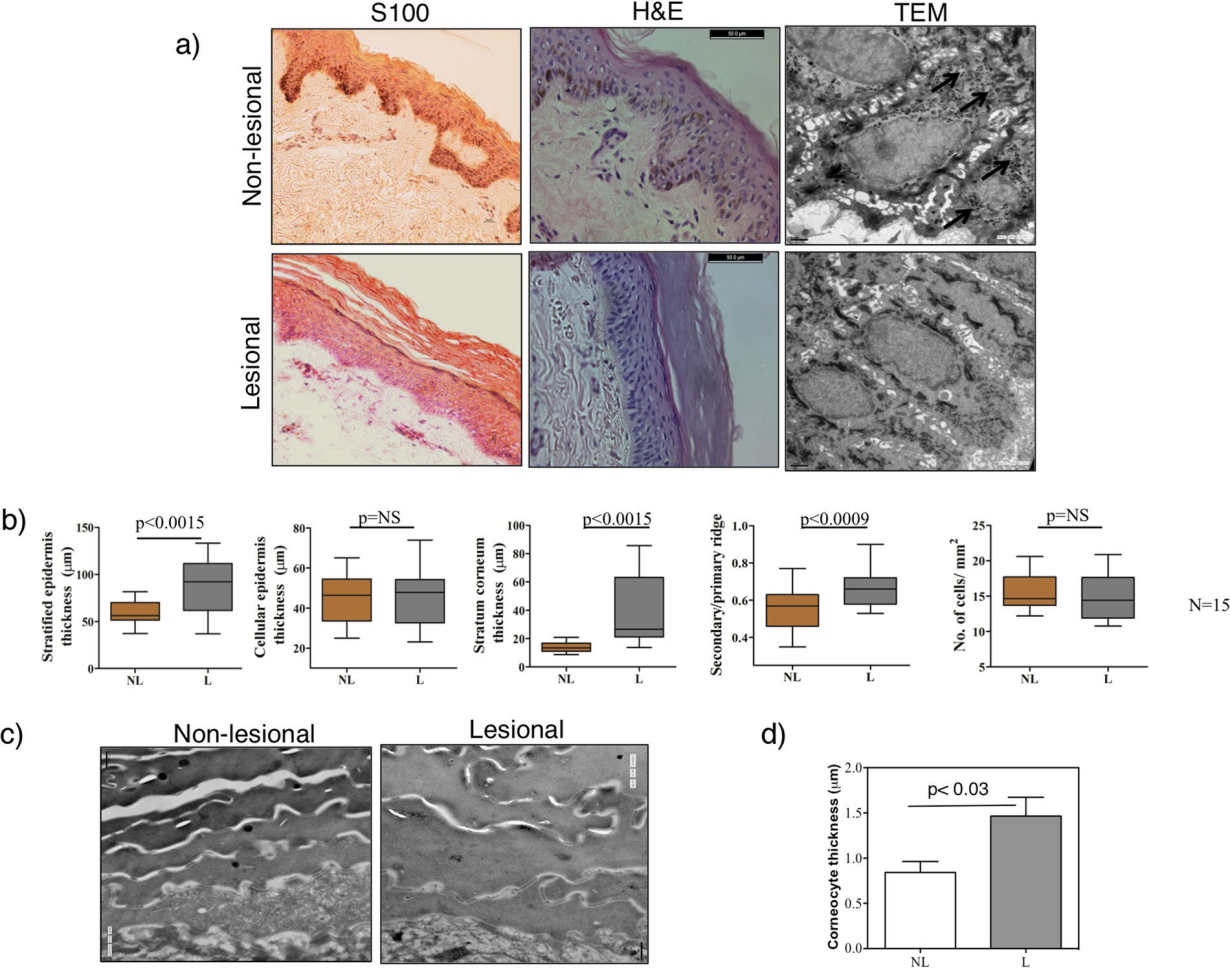
Architectural alterations in stable vitiligo lesions. (**a**) Immunohistochemical staining of melanocyte-specific S100 antigen, Hematoxylin and Eosin staining (H&E), and transmission electron micrographs (TEM) in paired non-lesional (NL) and lesional (L) skin sections. Arrows indicate melanosomes. Scale bar in S100 stained sections is 10 μm, 50 μm for H&E image and 1μm for TEM image. (**b**) Bar plots depicting architectural features quantitated in skin sections: thickness of stratified epithelia (from stratum basale to stratum corneum), cellular epidermis (from stratum basale to stratum granulosum) and thickness of stratum corneum in μm, ratio of length of secondary (μm) to primary ridge (μm) and the total number of cells per square mm in non-leisonal (NL) and lesional (L) samples (n = 15). The box plot represents the mean ± range of the data. Indicated p-values computed using a paired t-test involving n = 15 (NL vs L) pairs. (**c**) TEM images of stratum corneum (corneocytes) from non-lesional and lesional epidermis. Magnification is 1700X, scale bar is 0.5μm (**d**) Quantitation of thickness among three layers of corneocytes close to the viable epidermis across four pairs of matched NL and L skin sections, significance calculated using paired t test.

To investigate whether the transcriptome changes in structural and adhesion genes could modulate process of differentiation in keratinocytes, we performed histopathological studies of lesional and non-lesional skin. We obtained punch biopsies from matched lesional and non-lesional skin from 15 patients with stable non-segmental vitiligo. The biopsy samples were fixed and cryo-sectioned for staining with hematoxylin-eosin (H&E) or with anti-S100 antibody. As anticipated, S100-positive cells were found only in the non-lesional skin but not in the lesional skin indicating specific absence of melanocytes (Fig. 2a). Transmission electron microscopy (TEM) also revealed presence of melanosomes in the non-lesional epidermal keratinocytes but not in the lesional epidermis (Fig. 2a). The H&E staining pattern revealed that the lesional samples have a thickened stratum corneum and also the complete epidermis region (Fig. 2a). The data from these stained cryo-sections (n = 15 matched pairs) were quantified using imageJ software suite^23^ (Fig. 2b, Supplementary Fig. S3). We observed significant differences in the thickness of the stratified epithelia, from stratum basale to the top of stratum corneum in non-lesional and lesional skin samples (paired t-test, p < 0.0015). This thickening could be attributed to a more than two-fold increase (paired t test, p < 0.0015) in the thickness of stratum corneum in the lesional epidermis. Thickness of other viable cellular layers was comparable between the non-lesional and lesional samples (Fig. 2b). This is consistent with earlier studies that reported alterations in the thickness of stratum corneum and the whole epidermis in the lesional skin of vitiligo^24, 25^.

Careful examination of the H&E stained sections showed few additional architectural changes including majorly the blunting of the epidermis (Fig. 2a). We measured the epidermal thickness that extends from the epidermis, called rete ridges by calculating the ratio of the primary ridge (longest projection extending from stratum granulosum to stratum basale) to the secondary ridge (smallest projection extending from stratum granulosum to stratum basale). Interestingly, while the non-lesional epidermis showed secondary to primary ridge ratio far less than 1, this ratio in the lesional epidermis was closer to 1 confirming blunting of rete ridges in vitiligo lesions (Fig. 2b, paired t-test p < 0.0009). These changes seemed to be rather specific, since measurement of the cell packing density (cell numbers per unit area) did not reveal a significant difference between the non-lesional and lesional epidermis (Fig. 2b).

To rule out the possibility of anatomical differences contributing to the epidermal thickness in vitiligo (Table 1), we obtained seven pairs of matched lesional and non-lesional skin biopsies from anatomical sites proximal (P) to the lesional skin (within 5 cm distance from the margin of the lesions) and carried out histopathological evaluation. Statistical analyses revealed that the thickness of the stratified epidermis and that of the stratum corneum, as well as the rete ridge blunting were significantly different in the lesional skin compared to its neighboring non-lesional (proximal) skin (Supplementary Figs S4 and S5), providing further evidence that the thickened epidermis is indeed linked to vitiligo manifestation.

The increased thickness of the stratum corneum could be possibly due to an increase either in the total number of cornified layers or the size of the corneocytes that constitute the stratum corneum layer, or both. To address this, skin sections were subjected to TEM analysis. While taking ultrathin sections, we ensured that the stratum corneum layers were preserved. By measuring the corneocyte thickness in three consecutive corneum layers just above the stratum granulosum layer, our studies revealed larger corneocytes in the lesional skin (p < 0.03, n = 4) as compared to the non-lesional stratum corneum (Fig. 2c,d **)**. Together with the expression studies, this data strongly suggests that larger corneocytes probably compensates for the decreased expression of cornification components and thereby provide a thicker stratum corneum in vitiligo lesions.

### Putrescine levels are elevated in the lesional vitiligo epidermis

To delineate possible mechanisms underlying the formation of thickened stratum corneum in lesional epidermis, we examined pathways that can alter keratinocyte differentiation process. Intriguingly, transgenic mice overexpressing SSAT-1, which encodes spermine/spermidine acetyl transferase enzyme, was shown to have thicker stratum corneum with marked alterations in the keratinocyte differentiation process^26^. SSAT-1 catalyzes a key rate-limiting step in the degradation of spermidine (SPD) and spermine (SPN) to putrescine (PUT) and thus modulates polyamine levels. Polyamines are abundant metabolites present in all cell types and are known to participate in variety of cellular functions^27, 28^. Interestingly, an early study suggested enhanced polyamine-mediated crosslinking in the psoriatic skin^29^.

**Figure 3:**
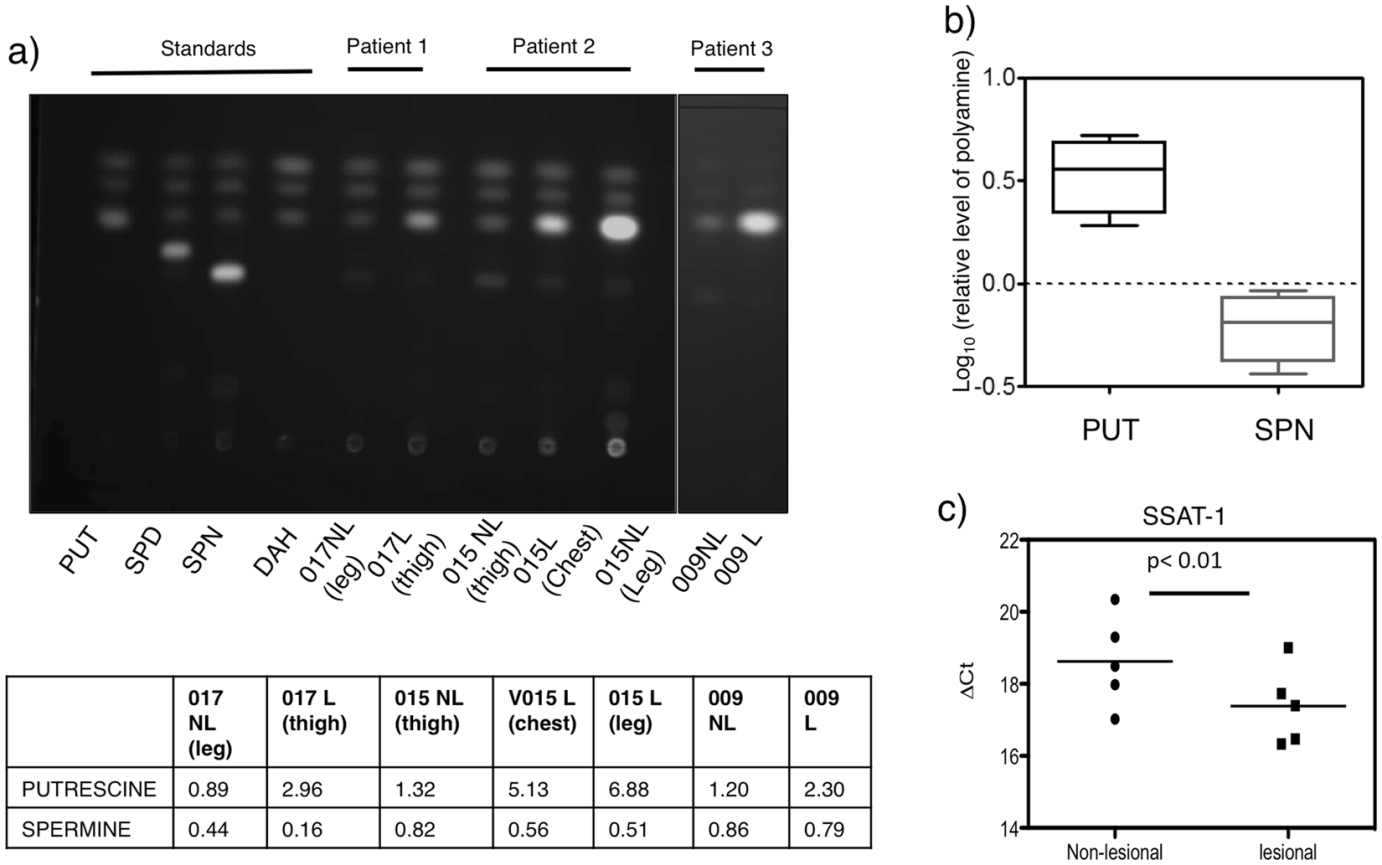
Altered polyamine metabolism in vitiligo epidermis. (**a**) Thin layer chromatographic analysis of polyamines in non-lesional and lesional epidermis after dansylation. Whole epidermis was extracted using perchloric acid and the extracts were dansylated along with standard polyamines. The reactions were extracted with toluene and spotted onto silica TLCs and developed on a cyclohexane: ethylacetate solvent system and detected under UV transilluminiscence. The amount of each of the polyamine per milligram of the epidermis is quantitated and represented across three patient samples (3 non-lesional and 4 lesional epidermis). (**b**) Log transformed relative levels of putrescine (PUT) and spermine (SPN) in three patient samples (3 non-lesional and 4 lesional epidermis) as detected by thin layer chromatography. Dotted line represents unchanged levels of polyamines with respect to the non-lesional skin (**c**) Real-time PCR analysis of Spermine/spermidine acetyl transferase-1 (SSAT-1) mRNA across five independent pairs of vitiligo lesional and non-lesional skin, represented as a scatter plot, horizontal line indicates mean. PUT- putriscine, SPD- spermidine, SPN- spermine, DAH- 1,7-diaminoheptane. NL- non-lesional, L- lesional skin.

We therefore examined whether lesional vitiligo skin possess an abnormal polyamine metabolism. We extracted polyamines from the epidermis of vitiligo subjects using perchloric acid extraction method^30^. Thin layer chromatographic analysis of polyamines showed an elevated level of PUT and a reduced level of SPN in lesional epidermis compared to non-lesional samples (Fig. 3a,b). The increased steady state level of PUT could either be an outcome of increased biosynthesis or could result from the breakdown of SPN and SPD (Supplementary Fig. S6). The reciprocal relationship between the level of PUT and SPN could potentially be linked to catabolism of SPN and SPD. Therefore to test ﻿this possibility, we measured SSAT-1 mRNA levels by real time qPCR and found ~3-fold elevated SSAT-1 mRNA level in lesional samples **(**Fig. 3c, p < 0.01, n = 5) suggesting that an increased SSAT-1 enzyme levels could lead to an enhanced polyamine catabolism in vitiligo. In most mammalian cells, SPN is the abundant polyamine and the levels of PUT are comparatively lower. However, higher levels of PUT compared to SPN in the skin and its further elevation in vitiligo lesions prompted elucidation of the specific role of PUT.

Corneocyte formation is an unusual differentiation process, wherein the cells undergo non-classical apoptosis^31^. The cells progressively lose the organelles but remain biochemically active. Structural proteins such as filaggrin and late cornified envelope proteins (LCEs) are extensively crosslinked by transglutaminase enzyme, giving rise to the corneocyte. As this layer showed extensive changes in lesional epidermis histologically and microarray analysis also demonstrated alterations in cornification process, we investigated whether the PUT was localized to the stratum corneum. Towards this, we specifically isolated the stratum corneum from normal epidermis using urea extraction protocol that dissolves the cellular epidermis. The isolated cornified layer was divided into two equal fractions: one was treated with perchloric acid for extracting non-covalently bound polyamines and the other was subjected to total acid hydrolysis (HCl) to obtain covalently conjugated polyamines from this tissue. We observed higher levels of PUT upon total acid hydrolysis, while other polyamines including SPN could not be detected from the stratum corneum, indicating the presence of covalently conjugated PUT in the cornified envelope (Supplementary Fig. S7). As the levels of PUT are elevated in the total epidermis of vitiligo lesions, we propose that these are localized to the stratum corneum layer and contribute to the alterations observed in the histological sections.

### Functional gene networks are altered in vitiligo

Having studied the architectural changes and their underlying molecular basis, we decided to address the implications of global transcriptional alterations observed in vitiligo. Only a small number of regulated genes belonged to recognizable categories in the enrichment analysis. However, the pathological manifestations observed in the vitiligo epidermis would be contributed by many of the regulated genes that form the vitiligo transcriptome. Since genetic predisposition is known in vitiligo and variants mapping to genes involved in different aspects of the disease are involved in the pathogenesis, we set out to address whether the altered gene expression profile in vitiligo lesions had any association with implicated genes. Towards this systems-level understanding, we constructed functional interactomes of genes that have been previously reported to genetically predispose individuals to vitiligo, in the genome-wide association studies (GWAS)^32^. A set of 45 genes associated with vitiligo served as the input for this analysis (Supplementary Table 2). The catalogue of physical/binding interactions for each of the 45 genes was extracted from the BIOGRID database^33^. This generated a correlated collection of global networks and sub-networks from the genes and their corresponding functional linkages i.e., interacting genes (Supplementary Fig. S8).

**Figure 4:**
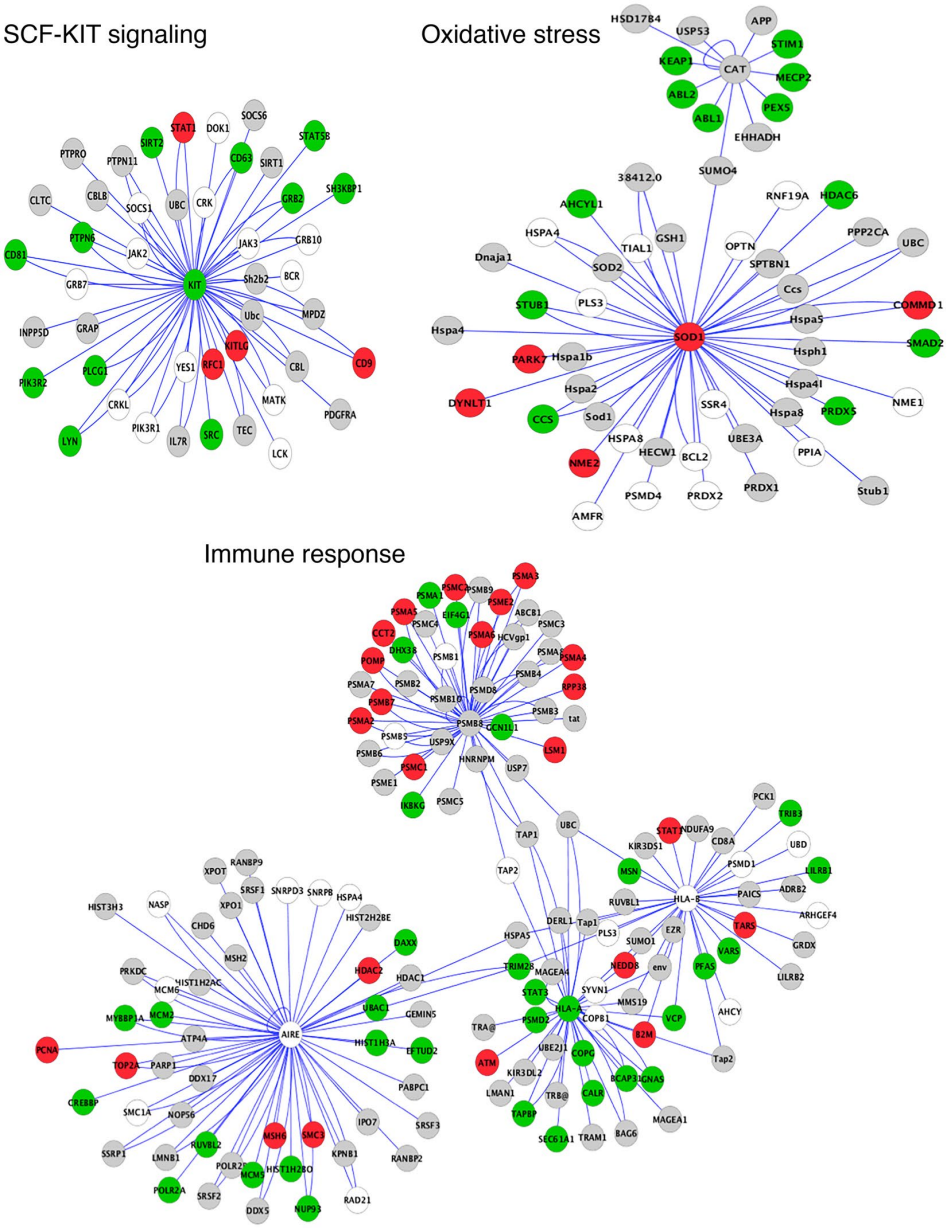
Altered functional networks in vitiligo. Network map of clusters of genes associated with vitiligo predisposition along with their interacting partners were mapped to the differential expression of genes observed in vitiligo. Three networks are shown: SCF-KIT signaling, oxidative stress and immune response. Each node (gene) is colored according to upregulation (red), downregulation (green), or no regulation (grey). White denotes proteins for which corresponding probes were not found in the microarray. The regulation score was calculated from microarray experiments performed on 15 vitiligo samples, where only agreement between >= 11 samples was included as a significant regulation.

Further, genes showing altered expressions in vitiligo microarray data were mapped onto the aforesaid networks. This second layer enabled us to understand “regulatory profiles” to reveal key perturbations in vitiligo. We analyzed the data by comparing changes in the number of subjects in whom a particular gene was differentially expressed in vitiligo lesions and was coded red or green to depict upregulation or downregulation respectively in at least 66% of the subjects analyzed (i.e. ≥11 out of 15 subjects). The network analysis implicated major perturbations in cellular processes such as SCF-KIT signaling, oxidative stress, apoptosis, stress response, vitamin D receptor and immune response (Fig. 4, Supplementary Fig. S9). Through this analysis we observed that the dynamic changes in RNA map to the same gene networks that are implicated in genetic predisposition of vitiligo. These networks link the functional state of skin in vitiligo to the known genetic predispositions in this complex disorder and provide an opportunity to comprehend the disease pathogenesis from the static genetic polymorphisms to dynamic gene regulatory networks.

## Discussion

Our study reports widespread alterations in the lesional epidermis at architectural, cellular, and transcriptomic levels and suggests an altered state of the epidermis in vitiligo. Despite heterogeneity in the appearance of clinical features between each vitiligo subject, an important observation that emerged from the genome-wide transcriptome analysis was the large-scale concerted changes in the cornification process in all 15 subjects with stable vitiligo. While the apparent downregulation of genes involved in melanogenesis is anticipated owing to the loss of melanocytes, alteration in keratinocyte-specific process is non-intuitive. We speculate that the absence of melanocytes from the vitiligo skin patch induces localized constraints for the maintenance of tissue homeostasis resulting in an altered state of the skin in vitiligo lesions.

Earlier transcriptomic studies on vitiligo have evaluated changes in intact biopsies containing both epidermis and the underlying dermis^18^. In that study, the authors also compared the lesions with the skin of healthy controls and provided important insights into the role of innate immune system and Natural Killer (NK) cells in causing melanocyte death. Another study on cultured human melanocytes from non-lesional skin of vitiligo subjects compared to healthy donors demonstrated abnormalities in melanocyte functions that could lead to antigen presentation by these cells^19^. In a recent genome-wide association study, altered Wnt signaling pathway was linked to melanocyte loss in vitiligo^34^. These studies have provided insights into different aspects of the disease biology. However, it is interesting to note that we still do not understand how the lesional skin maintains its functions and adapts to the absence of melanocytes. By analyzing matched lesional and non-lesional epidermal samples, we have not only minimized inter-individual differences but also provide a better understanding of alterations present in lesional epidermis, over and above the systemic alterations in vitiligo.

During our investigation, we observed widespread architectural differences in the lesional skin, as compared to the non-lesional skin. While changes in the thickness of epidermis were previously reported, we indicate that the thickening of epidermis is primarily due to the stratum corneum layer. We also highlight the blunting of rete-ridges at the junction of epidermis and dermis, which would decrease surface area of interaction in the lesional skin. Further, our studies demonstrate enlarged corneocytes in the lesions, which contribute to the thickening of the stratum corneum. The lesional skin is seemingly functional as there are no apparent changes in the skin barrier reported clinically in vitiligo subjects.

On a mechanistic basis, the molecular change contributed by polyamine metabolism is a new perspective unraveled in this study that could potentially explain several changes observed in the lesional vitiligo skin. Polyamines contribute to various cellular functions and their role in skin is addressed mainly in the context of cancers^35^. Our study demonstrates upregulation of SSAT-1, the key rate-limiting enzyme involved in polyamine catabolism with a concomitant increase in PUT and a decrease in SPN. While in most cells polyamines have a conventional role, skin is different in having abundance of PUT, which we could trace to the stratum corneum layers in the normal healthy skin. It is tempting to speculate that the lesional epidermal PUT in vitiligo contributes to the thicker stratum corneum. In addition to changes in keratinocyte differentiation brought about by altered polyamine levels, PUT could be involved in structural changes by participating in covalent couplings as we observe far greater levels of PUT upon hydrolysis compared to perchloric acid extraction. We hypothesize that the thicker stratum corneum in vitiligo skin is a likely adaptation that probably protects vitiligo keratinocytes from ultraviolet radiations. Associated changes in skin architecture such as the epidermal blunting could be an outcome of alterations in the proliferative capacity of the stem cell niche, which needs to be further evaluated.

Mapping of vitiligo transcriptomic changes onto the interactome network generated from the known genetic associations enabled us to visualize the dynamic changes in the genome that could culminate in vitiligo. It is interesting to note that in most categories the key implicated node is not affected but the associated genes show concerted changes, which indicates alteration in the functioning of that pathway. c-KIT mediated signaling is critical in maintaining the melanocyte survival and it is interesting that the entire network is down-regulated. Though SCF (KITLG) is upregulated, a decrease in downstream components would render this pathway dysfunctional. In the immune system module, proteosomal components are upregulated supporting the role of antigen presentation and autoimmunity in vitiligo. Further studies on these pathways would help to delineate their role in maintaining the diseased state and altered homeostasis in vitiligo and offer interventions for correction.

Besides the apparent depigmentation, the altered lesional epidermis is competent to perform its biological functions and is also suggested to be protective against skin cancers^4, 5^. We propose that architectural, cellular and molecular changes observed in this study may be playing a protective role against carcinogenic UV-induced damage. Such adaptive changes in physiological conditions have been defined as ‘enantiostatic’ adaptations^36, 37^. While biological systems have a tendency to revert back to homeostatic state, vitiligo skin is a challenge in terms of restoring tissue function due to the permanent loss of melanocytes. The adaptations described in our study whereby the system adapts itself to maintain the essential function is an example of tissue ‘enantiostasis’. Such modifications while on one hand may be protective response to maintain the integrity of the tissue, but on the other hand could contribute to the maintenance of disease states. Such changes in tissue modification may be a contributory factor in variety of other pathological conditions. Future studies would focus on finding ways to reverse these enantiostatic states that could provide means to treat stable vitiligo patches.

In summary, our study provides important insights into the pathophysiology of vitiligo skin and delineates gross tissue level perturbations of the epidermis in vitiligo. Many of these changes could be adaptive in nature and may be involved in protecting the skin from environmental insults in the absence of melanin. We propose that these adaptive changes also preclude remigration and sustenance of melanocytes from neighboring uninvolved skin. Currently stable vitiligo is treated by enriched stem cell pool of melanocytes with limited success. Strategies that can incorporate restoration of architectural niche of vitiligo skin may greatly improve autologous transplantation therapy.

## Materials and Methods

### Patient recruitment and sampling

Skin punch biopsies (3–4 mm) from lesional and non-lesional sites from vitiligo patients were obtained after taking informed consent. Institutional Human ethics committees of Ram Manohar Lohia Hospital, New Delhi and National institute of Immunology, New Delhi, approved the study and it is in agreement with Declaration of Helsinki principles. Non-lesional biopsies were obtained from gluteal/lateral thigh region and the lesional samples were taken from affected parts of the body (Table 1). All the lesional and non-lesional skin biopsies were obtained from stable vitiligo vulgaris patients scheduled to undergo punch grafting or melanocyte/epidermal cell transplantation. The biopsies were obtained from the patients prior to therapeutic surgical intervention. The proximal non-lesional biopsies were obtained from non-lesional sites within a distance of 5 cm from the margins of the lesions. All the patients included in the present study were Indians, had non-segmental vitiligo (vitiligo vulgaris), and the disease was stable for at least 6 months at the time of taking the biopsy.

### Quantitation of architectural changes in vitiligo

Punch biopsies were immediately fixed in buffered formalin solution and four-micron thick cryo-sections were stained with hematoxylin and eosin. Morphometric evaluation of the images was carried out using ImageJ software and the data was analyzed using paired t-test using Graphpad Prism suite and represented as mean ± range of the measurement. For detecting melanocytes, sections were stained with S100 antibody (dilution 1:100; Dako Cytomation, Glostrup, Denmark) for 45 minutes at room temperature. Slides were washed and incubated with labeled polymer alkaline phosphatase (Dako Cytomation) for 30 minutes at room temperature. Reaction was developed with fuchsin counterstained with hematoxylin.

### RNA isolation and microarray

Total RNA was isolated using Trizol method from the whole epidermal cells from 15 pairs of non-lesional and lesional skin samples after separation of epidermis from the dermis using Dispase II. RNA samples were cleaned using RNEasy columns form Qiagen. RNA integrity and quality was measured using Bioanalyzer and samples with RIN score ≥ 8 were taken ahead for labelling and hybridization. Whole genome microarray was carried out using Illumina WG-6 array using manufacturer’s guidelines. Preliminary data normalization and analysis was carried out using Bead Studio software. Further analysis was carried out using Genome Studio version 2011.1. High throughput data of 48,803 probes specific for 37804 genes from non-lesional and vitiligo epidermis of 15 subjects was thus obtained.

### Vitiligo data analysis

The probes with background subtracted expression values of genes in negative were eliminated from the analysis and the expression values were average normalized. This was done by taking the mean of the entire data for each of the 15 NL and L samples, dividing every expression value by this mean and then multiplying it by 500. Paired t-test was carried out using Matlab (Mathworks, Inc, Natick, MA).

### Transmission electron microscopy

Transmission electron microscopy was performed on non-lesional and lesional vitiligo skin samples using standard methods. Briefly, 3 mm skin pieces were fixed in 2.5% gluteraldehyde and 4% paraformaldehyde, osmicated in 1% osmium tetroxide, dehydrated in graded series of alcohol and infiltrated with Epon 812 resin. Ultrathin sections were cut on RMC ultramicrotome, collected on copper grids and stained with uranyl acetate and lead citrate. Samples were visualized on Tecnai G2 20 twin (FEI) transmission electron microscope.

### TLC for Polyamine detection

Whole epidermis was dissolved in 2% perchloric acid and dansylated using 400 μl dansyl chloride (5 mg/ml) at pH 2.0. 200 μl of saturated sodium carbonate was added and incubated at 70 °C for 10 min and the reaction was quenched using 100 μl of 150 mg/ml proline at room temperature for 30 min. Dansylated polyamine was extracted using 500 μl Toluene and spotted on silica thin layer chromatographic column along with standard dansylated polyamines. The mixture was separated using cyclohexane ethyl acetate solvent system and visualized under UV transilluminence (500 millisec exposure). PUT, SPD, SPN in first 3 lanes were used as standards. DAH was used as internal standard to calculate the relative amount of specific polyamines present in the epidermis with respect to corresponding standards. Image Quant 5.2 software was used for densitometry quantification.

### Functional networks generation

An initial dataset of 45 genes showing predisposition to vitiligo was obtained from previously characterized GWAS studies^32^. Protein interactions for each gene was prepared using BIOGRID database^33^. This database stores human protein interactions that have been experimentally characterized and therefore, served as a good repository for in-house generated vitiligo protein network. In total, we obtained 1416 interactions from input set of 45 genes (or proteins). We prepared a network map using Cytoscape, a commonly used visualization tool to construct linkages between proteins. The global network resulted in six major sub-networks (hubs with densely connected proteins), namely, KIT, XBP1-MSH6, SOD-CAT, PSMB8-AIRE-HLA, FAS-CASP, and VDR as shown in Supplementary Fig. S8. The physical interactions of the proteins depicted in the 6 sub-networks are shown in Supplementary Table 3. These sub-networks were then overlaid with vitiligo microarray expression information with each mRNA (protein) showing upregulation (red), downregulation (green), or no regulation (grey). The regulation score was calculated from microarray experiments performed on 15 vitiligo samples, where similar regulation in ≥11 samples was considered as a significant regulation. Thus, Fig. 4 and Supplementary Fig. S9 shows a comprehensive functional interactome of protein-interaction and their regulation patterns of the 6 sub-networks in vitiligo subjects.

### Data and materials availability

Microarray data has been deposited in NCBI Gene Expression Omnibus portal accession number GSE75819.

## Electronic supplementary material

Supplementary figures and tables

## References

[1] Cichorek M, Wachulska M, Stasiewicz A, Tyminska A (2013). Skin melanocytes: biology and development. Postepy Dermatol. Alergol.

[2] Natarajan VT, Ganju P, Ramkumar A, Grover R, Gokhale RS (2014). Multifaceted pathways protect human skin from UV radiation. Nat. Chem. Biol..

[3] Brenner M, Hearing VJ (2008). The protective role of melanin against UV damage in human skin. Photochem. Photobiol.

[4] Schallreuter KU, Tobin DJ, Panske A (2002). Decreased photodamage and low incidence of non-melanoma skin cancer in 136 sun-exposed caucasian patients with vitiligo. Dermatology.

[5] Teulings HE (2013). Decreased risk of melanoma and nonmelanoma skin cancer in patients with vitiligo: a survey among 1307 patients and their partners. Br. J. Dermatol.

[6] Falabella R, Barona MI (2009). Update on skin repigmentation therapies in vitiligo. Pigment Cell Melanoma Res.

[7] Moretti S (2009). Keratinocyte dysfunction in vitiligo epidermis: cytokine microenvironment and correlation to keratinocyte apoptosis. Histol. Histopathol..

[8] Montes LF, Abulafia J, Wilborn WH, Hyde BM, Montes CM (2003). Value of histopathology in vitiligo. Int. J. Dermatol..

[9] Panuncio AL, Vignale R (2003). Ultrastructural studies in stable vitiligo. Am. J. Dermatopathol..

[10] Moellmann G, Klein-Angerer S, Scollay DA, Nordlund JJ, Lerner AB (1982). Extracellular granular material and degeneration of keratinocytes in the normally pigmented epidermis of patients with vitiligo. J. Invest. Dermatol..

[11] Hann SK, Park YK, Lee KG, Choi EH, Im S (1992). Epidermal changes in active vitiligo. J. Dermatol..

[12] Passi S, Grandinetti M, Maggio F, Stancato A, De Luca C (1998). Epidermal oxidative stress in vitiligo. Pigment Cell Res.

[13] Rokos H, Beazley WD, Schallreuter KU (2002). Oxidative stress in vitiligo: photo-oxidation of pterins produces H(2)O(2) and pterin-6-carboxylic acid. Biochem. Biophys. Res. Commun..

[14] Schallreuter KU (1999). Successful treatment of oxidative stress in vitiligo. Skin Pharmacol. Appl. Skin Physiol..

[15] Pretti Aslanian FM, Noe RA, Cuzzi T, Filgueira AL (2007). Abnormal histological findings in active vitiligo include the normal-appearing skin. Pigment Cell Res.

[16] Verma G, Varkhande SR, Kar HK, Rani R (2015). Evaluation of Repigmentation with Cultured Melanocyte Transplantation (CMT) Compared with Non-Cultured Epidermal Cell Transplantation in Vitiligo at 12th Week Reveals Better Repigmentation with CMT. J. Invest. Dermatol..

[17] van Geel N, Goh BK, Wallaeys E, De Keyser S, Lambert J (2011). A Review of Non-cultured Epidermal Cellular Grafting in Vitiligo. J. Cutan. Aesthet. Surg.

[18] Yu R (2012). Transcriptome analysis reveals markers of aberrantly activated innate immunity in vitiligo lesional and non-lesional skin. PLoS One.

[19] Stromberg S (2008). Transcriptional profiling of melanocytes from patients with vitiligo vulgaris. Pigment Cell Melanoma Res.

[20] Lee AY (2012). Role of keratinocytes in the development of vitiligo. Ann. Dermatol..

[21] Lee AY, Kim NH, Choi WI, Youm YH (2005). Less keratinocyte-derived factors related to more keratinocyte apoptosis in depigmented than normally pigmented suction-blistered epidermis may cause passive melanocyte death in vitiligo. J. Invest. Dermatol..

[22] Sandby-Moller J, Poulsen T, Wulf HC (2003). Epidermal thickness at different body sites: relationship to age, gender, pigmentation, blood content, skin type and smoking habits. Acta Derm. Venereol..

[23] Schneider CA, Rasband WS, Eliceiri KW (2012). NIH Image to ImageJ: 25 years of image analysis. Nat. Methods..

[24] Gniadecka M, Wulf HC, Mortensen NN, Poulsen T (1996). Photoprotection in vitiligo and normal skin. A quantitative assessment of the role of stratum corneum, viable epidermis and pigmentation. Acta Derm. Venereol..

[25] Jung SE, Kang HY, Lee ES, Kim YC (2015). Changes of epidermal thickness in vitiligo. Am. J. Dermatopathol..

[26] Pietila M (2005). Disturbed keratinocyte differentiation in transgenic mice and organotypic keratinocyte cultures as a result of spermidine/spermine N-acetyltransferase overexpression. J. Invest. Dermatol..

[27] Spotheim-Maurizot M, Ruiz S, Sabattier R, Charlier M (1995). Radioprotection of DNA by polyamines. Int. J. Radiat. Biol..

[28] Newton GL, Aguilera JA, Ward JF, Fahey RC (1996). Polyamine-induced compaction and aggregation of DNA–a major factor in radioprotection of chromatin under physiological conditions. Radiat. Res..

[29] Martinet N, Beninati S, Nigra TP, Folk JE (1990). N1N8-bis(gamma-glutamyl)spermidine cross-linking in epidermal-cell envelopes. Comparison of cross-link levels in normal and psoriatic cell envelopes. Biochem. J.

[30] Madhubala R (1998). Thin-layer chromatographic method for assaying polyamines. Methods Mol. Biol..

[31] Eckhart L, Lippens S, Tschachler E, Declercq W (2013). Cell death by cornification. Biochim. Biophys. Acta.

[32] Jin Y (2012). Genome-wide association analyses identify 13 new susceptibility loci for generalized vitiligo. Nat. Genet..

[33] Chatr-Aryamontri A (2015). The BioGRID interaction database: 2015 update. Nucleic Acids Res.

[34] Regazzetti C (2015). Transcriptional Analysis of Vitiligo Skin Reveals the Alteration of WNT Pathway: A Promising Target for Repigmenting Vitiligo Patients. J. Invest. Dermatol..

[35] Nowotarski SL, Woster PM, Casero RA (2013). Polyamines and cancer: implications for chemotherapy and chemoprevention. Expert. Rev. Mol. Med..

[36] Mangum C, Towle D (1977). Physiological adaptation to unstable environments. Am. Sci.

[37] Raubenheimer D, Simpson SJ, Tait AH (2012). Match and mismatch: conservation physiology, nutritional ecology and the timescales of biological adaptation. Philos. Trans. R. Soc. Lond. B. Biol. Sci..

