# Supplementary material for: Mapping architectural and transcriptional alterations in non-lesional and lesional epidermis in vitiligo
Source: Sci Rep. 2017 Aug 29;7:9860. doi: 10.1038/s41598-017-10253-w (PMC5575244; doi:10.1038/s41598-017-10253-w)
Supplement: Supplementary file 1 — Supplementary figures and tables [file 41598_2017_10253_MOESM1_ESM.pdf]

Mapping architectural and transcriptional alterations in non-lesional and lesional epidermis in Vitiligo

Archana Singh[1], Vishvabandhu Gotherwal[1,3], Päivi Junni[4], Vinaya Vijayan[5], Manisha Tiwari[1], Parul Ganju[2], Avinash Kumar[2], Pankaj Sharma[6], Tanveer Fatima[6], Aayush Gupta[7], Ananthaprasad Holla[8], Hemanta K. Kar[6], Sangeeta Khanna[1], Lipi Thukral[1], Garima Malik[1], Krishnamurthy Natarajan[9], Chetan J. Gadgil[5], Riitta Lahesmaa[4], Vivek T. Natarajan[1,3*], Rajni Rani[2*] and Rajesh S. Gokhale[1,2,5,10*]

Affiliations:

[1]CSIR-Institute of Genomics and Integrative Biology, Mathura Road, Delhi, India.

[2]National Institute of Immunology, ArunaAsaf Ali Marg, New Delhi, India.

[3]Academy of Scientific and Innovative Research, Delhi, India

[4]Turku Centre for Biotechnology, University of Turku and ÅboAkademi University, Finland.

[5]CSIR-National Chemical Laboratory, Chemical Engineering Division, Pune, India.

[6]Department of Dermatology, Post Graduate Institute for Medical Education and Research (PGIMER), Dr. Ram Manohar Lohia Hospital, New Delhi, India.

[7]DY Patil Medical College, Pimpri, Pune, India.

[8]MelanoSite, Center for Advanced Vitiligo Treatment and Collaborative Pigment Cell Research Delhi, India.

[9]School of Life Sciences, Jawaharlal Nehru University, New Delhi, India.

[10]Jawaharlal Nehru Centre for Advanced Scientific Research, Bangalore, India.

[*] Corresponding Author

Keywords: Vitiligo, transcriptome, putrescine, tissue remodeling, epidermis, cornification.

Running Title: Architectural and transcriptional alterations in vitiligo

Supplementary Figures and tables:

Figure S1: Unsupervised clustering of genes involved in maintaining epidermal architecture and cell-cell adhesion of keratinocytes. Blue is downregulated, and red is upregulated.

Figure S2: Transcriptional regulation of genes involved in keratinization and cornification process. Fold change values plotted for 6 lesional epidermis samples compared to their corresponding non-lesional tissue. Significance is calculated using paired t test (n=6). * indicates significant change in lesional skin. 18s rRNA was used as normalization control. SYBR green chemistry was used for Real time PCR.

Figure S3: Hematoxylin and Eosin (H&E) stained sections of Non-lesional (NL) and Lesional (L) skin of vitiligo subjects (N=15).

Figure S4: Hematoxylin and Eosin (H&E) stained sections of non-lesional (NL), lesional (L) and non-lesional skin proximal to the lesion (P) in vitiligo subjects (N=7).

Figure S5: Architectural alterations in lesional vs proximal non-lesional skin

Bar plots depicting architectural features quantitated in skin sections: thickness of stratified epithelia (from stratum basale to stratum corneum), cellular epidermis (from stratum basale to stratum granulosum) and thickness of stratum corneum in μm, ratio of length of secondary (μm) to primary ridge (μm) and the total number of cells per square mm (n=7 pairs) in lesional (L) and proximal (P) samples. 'P' refers to non-lesional skin biopsy taken from anatomical site proximal to the vitiligo lesion. The box plot represents the mean ± range of the data. Indicated p-values computed using a paired t-test involving n=7 pairs (L vs P).

Figure S6: Polyamine metabolism pathway: The three polyamines, putriscine, spermidine and spermine are derived from ornithine. SMS converts putriscine to spermidine and spermine, which are further converted by SSAT-1 enzyme to N1-acetyly spermidine and N1-acetylspermine respectively. Breakdown of the N-acetylated polyamines by PAOX results in putriscine. SSAT-1 expression is upregulated at transcriptional level in lesional skin (indicated in red color). ODC-ornithine decarboxylase, SRM-spermidine synthase, SMS- spermine synthase, SMO-spermine oxidase, SAT-1- PAO- peroxisomal N(1)-acetyl-spermine/spermidine oxidase, MAOB- monoamine oxidase B, SSAT1- spermidine/spermine N1-acetyltransferase 1, TGM1- transglutaminase 1.

Figure S7: Thin layer chromatographic analysis of polyamines in normal healthy skin (a) Thin layer chromatographic analysis of polyamines extracted using perchloric acid (non-covalently bound) or hydrolyzed using hydrochloric acid (covalently conjugated) from isolated stratum corneum from normal healthy skin. PUT, SPD, SPN in first 3 lanes were used as standards. DAH was used as internal standard to calculate the relative amount of specific polyamines present in the epidermis. PUT- Putrescine, SPD- spermidine, SPN- spermine, DAH- 1,7-diaminoheptane.

Figure S8: Global network of genes and its corresponding functional linkages i.e., interacting genes. Each protein is colored according to upregulation (red), downregulation (green), or no regulation (grey). White denotes proteins for which corresponding probes were not found in the microarray. The regulation score was calculated from microarray experiments performed on 15 vitiligo samples, where only agreement between >=11 samples was included as a significant regulation.

Figure S9: Altered functional networks in vitiligo

Network map of clusters of genes associated with vitiligo predisposition along with their interacting partners were mapped to the differential expression of genes observed in vitiligo. Three networks are shown- vitamin D receptor, apoptosis and stress response. Each node (gene) is colored according to upregulation (red), downregulation (green), or no regulation (grey). White denotes proteins for which

corresponding probes were not found in the microarray. The regulation score was calculated from microarray experiments performed on 15 vitiligo samples, where only agreement between >=11 samples was included as a significant regulation.

Supplementary Table 1: Top 10 differentially regulated pathways in genome-wide transcriptome analysis of non-lesional and lesional skin.

Supplementary Table 2: List of 45 genes used as input for constructing functional interactome.

Supplementary Table 3: List of protein-protein interactions for the six subnetworks depicted in Figure 4. These genes directly interact physically/ functionally with the genes that harbor predisposition to vitiligo (as listed in supplementary table 2). pi denotes physical interaction.

Fig S1

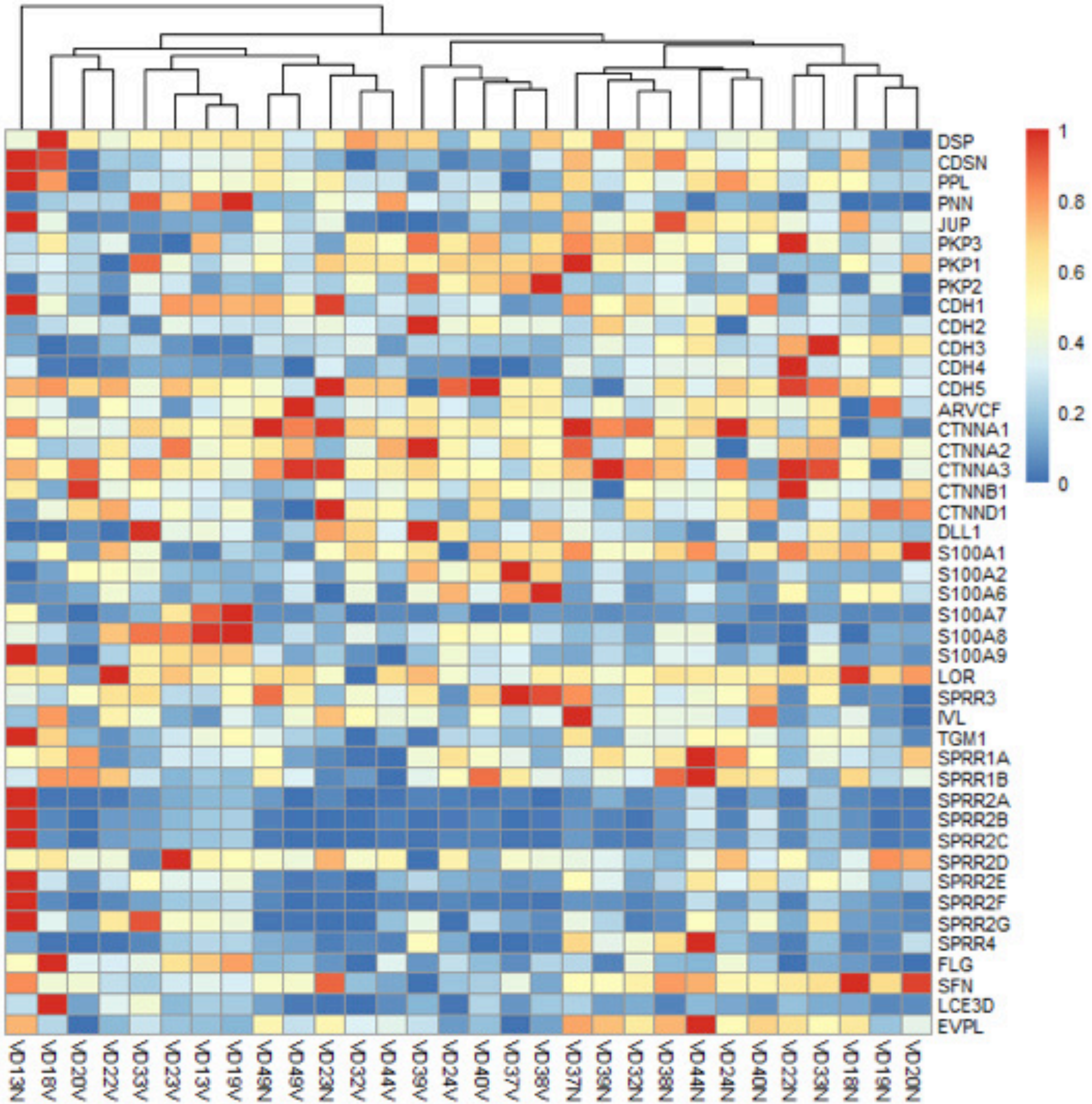
DSP
CDSN
PPL
PNN
JUP
PKP3
PKP1
PKP2
CDH1
CDH2
CDH3
CDH4
CDH5
ARVCF
CTNNA1
CTNNA2
CTNNA3
CTNNB1
CTNND1
DLL1
S100A1
S100A2
S100A6
S100A7
S100A8
S100A9
LOR
SPRR3
IVL
TGM1
SPRR1A
SPRR1B
SPRR2A
SPRR2B
SPRR2C
SPRR2D
SPRR2E
SPRR2F
SPRR2G
SPRR4
FLG
SFN
LCE3D
EVPL
VD13N
VD18V
VD20V
VD22V
VD33V
VD23V
VD13V
VD19V
VD49N
VD49V
VD23N
VD32V
VD44V
VD39V
VD24V
VD40V
VD37V
VD38V
VD37N
VD39N
VD32N
VD38N
VD44N
VD24N
VD40N
VD22N
VD33N
VD18N
VD19N
VD20N
1
0.8
0.6
0.4
0.2
0

Fig S2

Regulation of genes involved in keratinization and cornification process

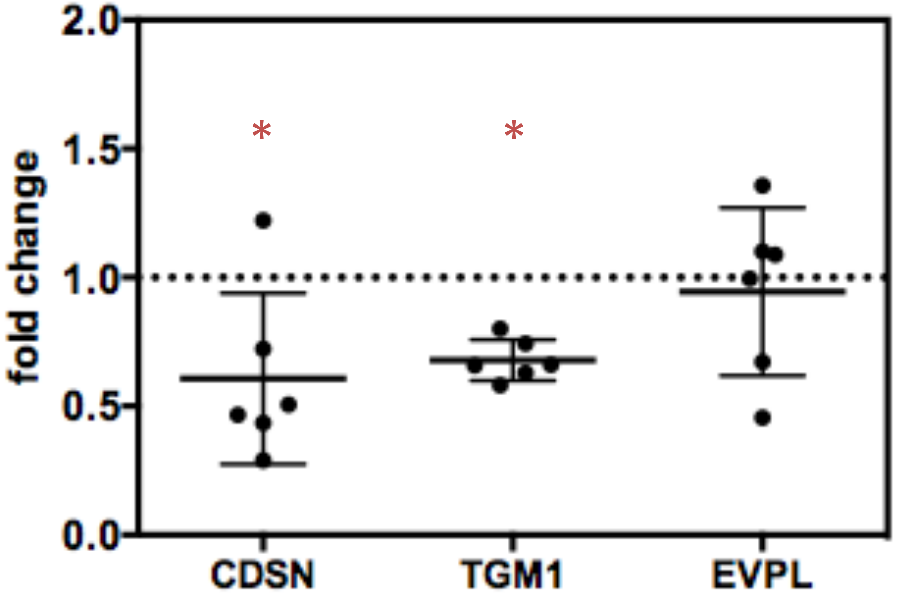

Fig S3

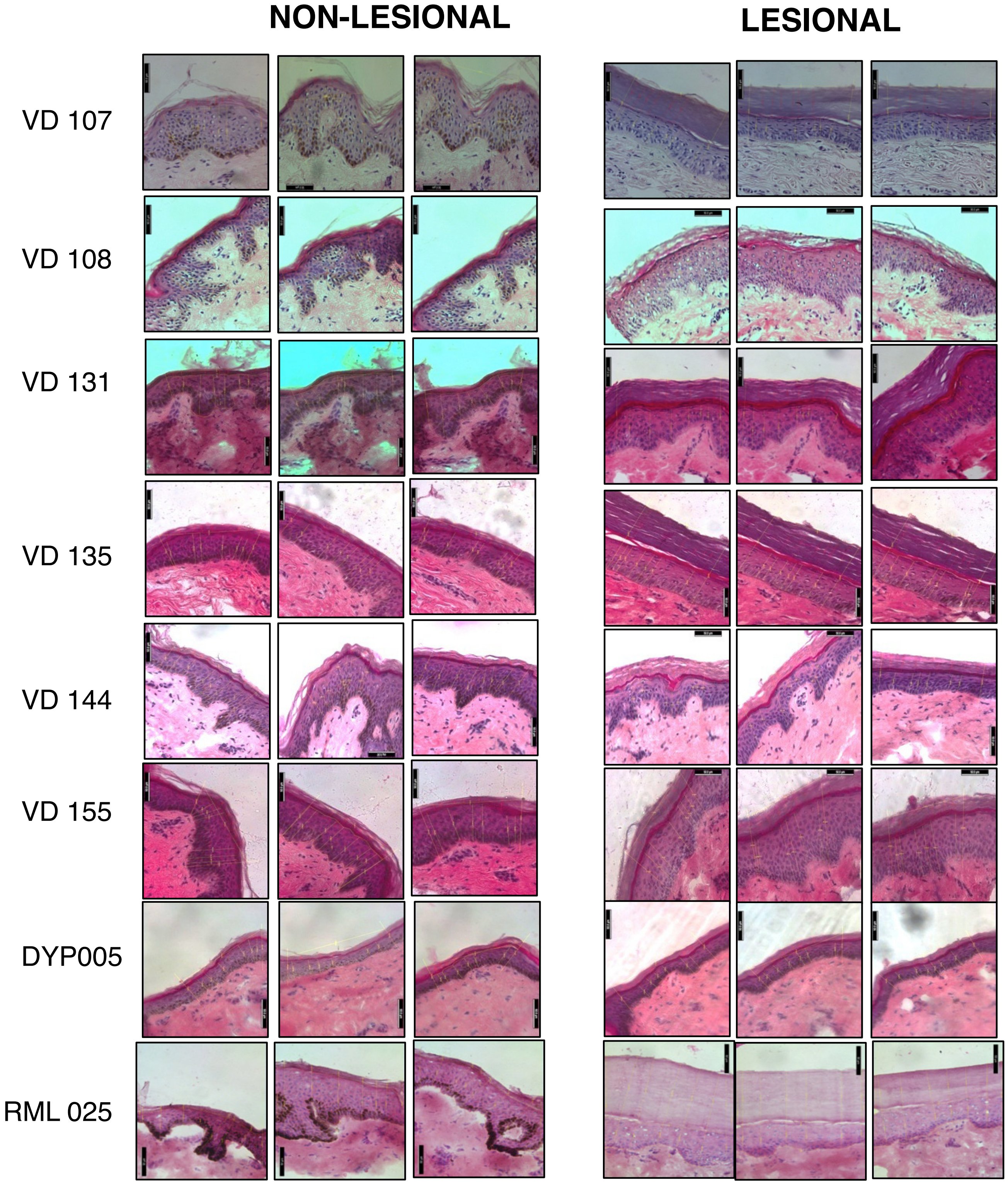
NON-LESIONAL
LESIONAL
VD 107
VD 108
VD 131
VD 135
VD 144
VD 155
DYP005
RML 025

Fig S4

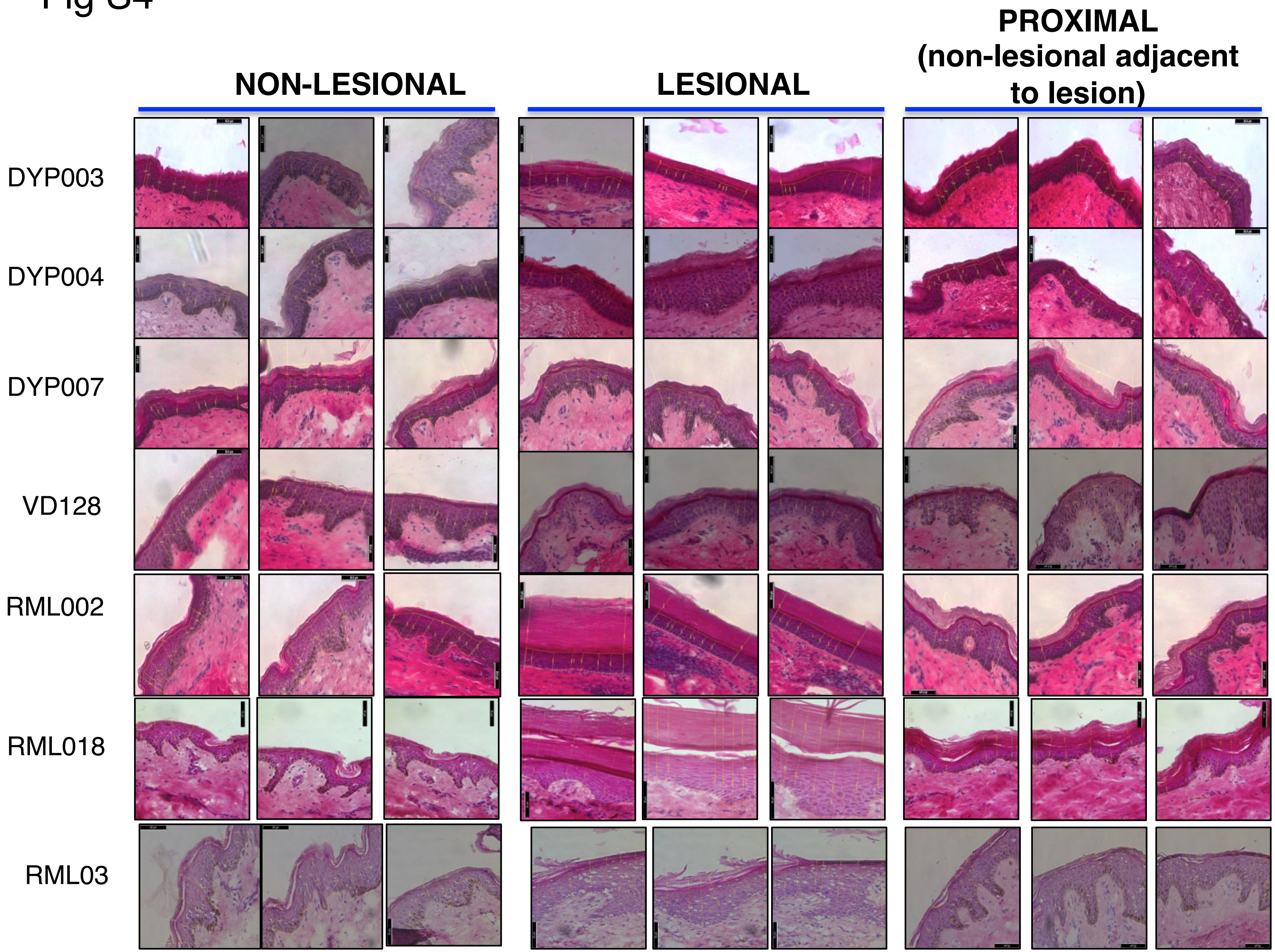
NON-LESIONAL
LESIONAL
PROXIMAL
(non-lesional adjacent
to lesion)
DYP003
DYP004
DYP007
VD128
RML002
RML018
RML03

Fig S5

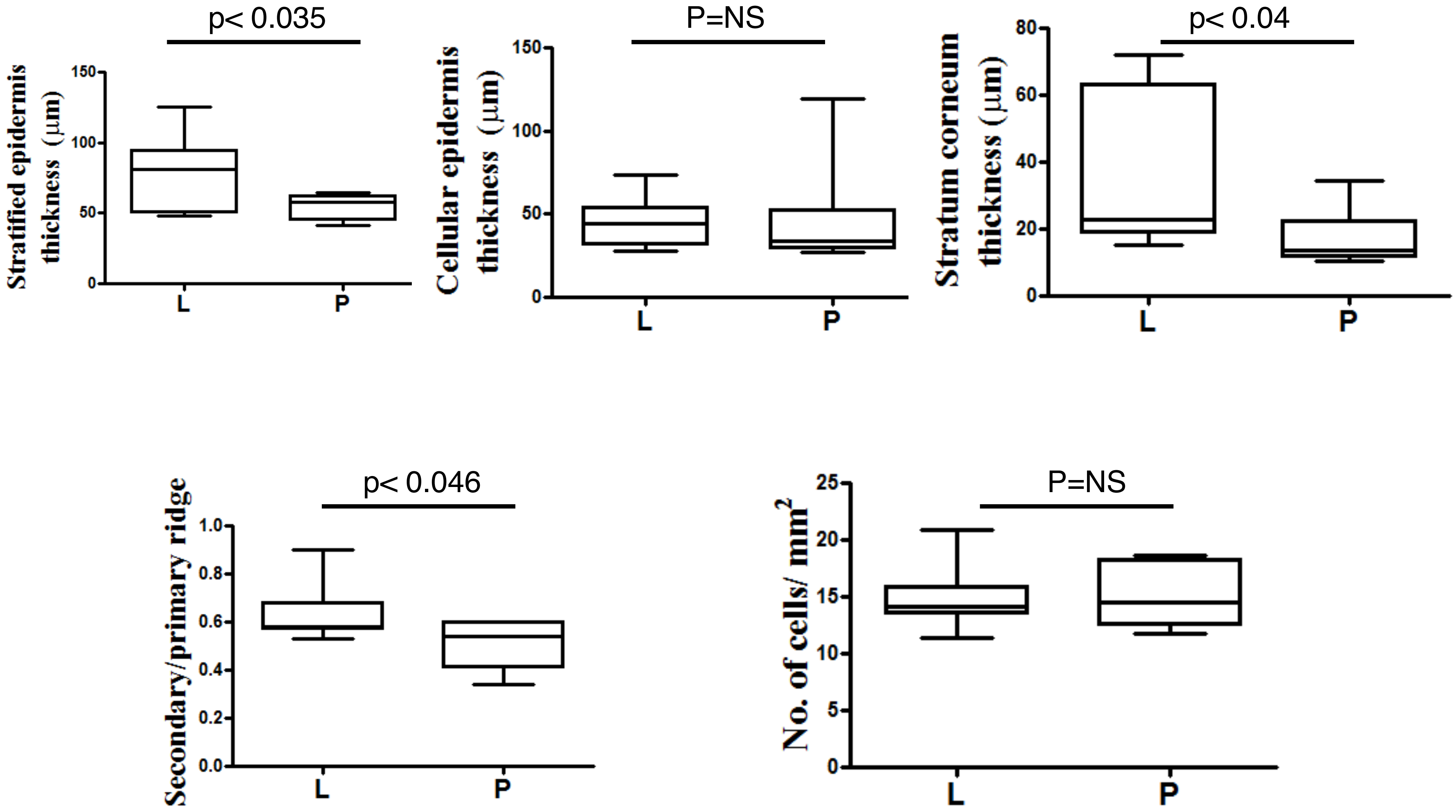
p< 0.035
Stratified epidermis thickness (µm)
150
100
50
0
L
P
P=NS
Cellular epidermis thickness (µm)
150
100
50
0
L
P
p< 0.04
Stratum corneum thickness (µm)
80
60
40
20
0
L
P
p< 0.046
Secondary/primary ridge
1.0
0.8
0.6
0.4
0.2
0.0
L
P
P=NS
No. of cells/ mm2
25
20
15
10
5
0
L
P

Fig S6

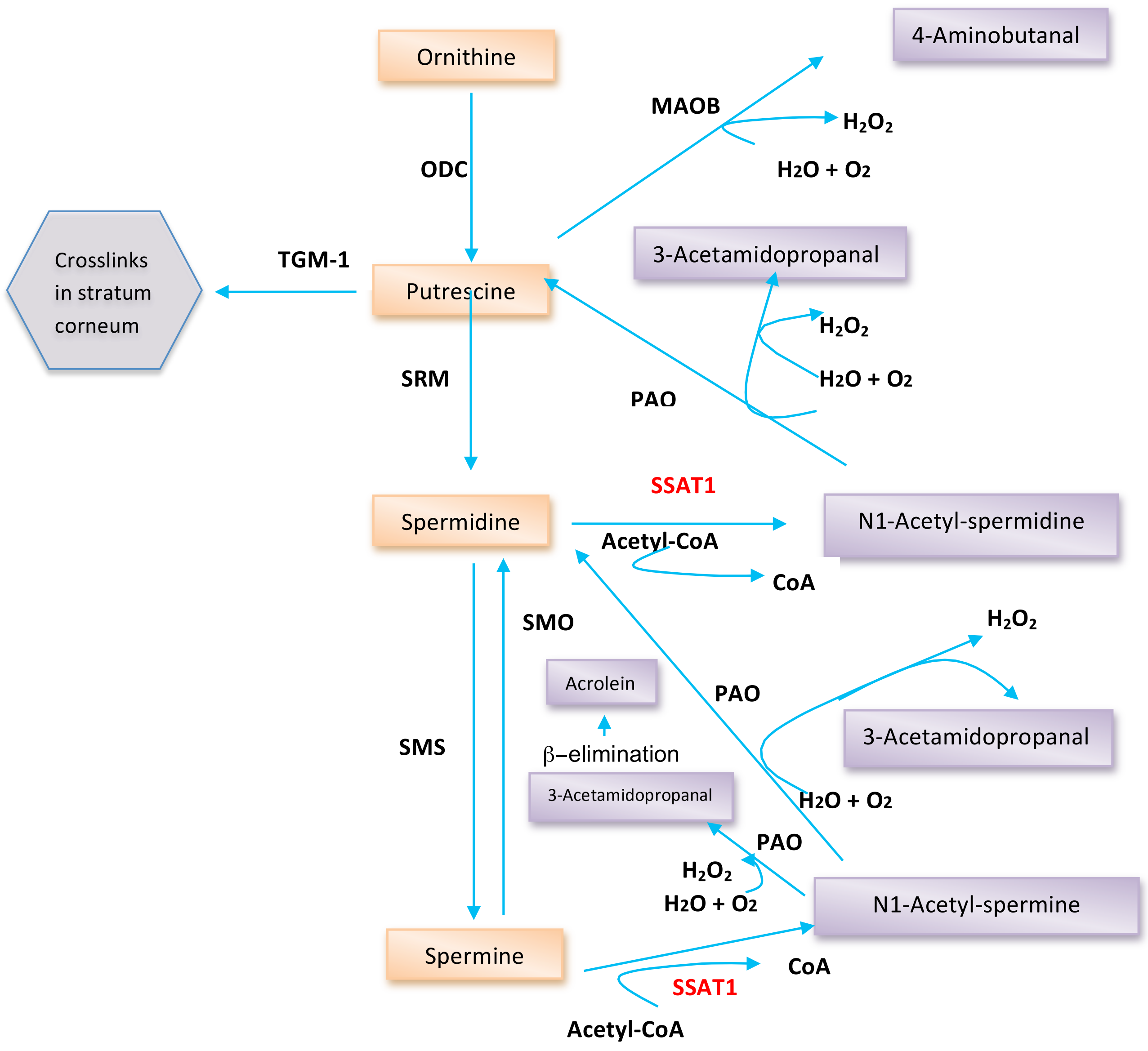
Ornithine
ODC
Putrescine
TGM-1
Crosslinks in stratum corneum
MAOB
H_2O_2
$H_2O + O_2$
4-Aminobutanal
3-Acetamidopropanal
H_2O_2
$H_2O + O_2$
PAO
SRM
Spermidine
SSAT1
Acetyl-CoA
CoA
N1-Acetyl-spermidine
SMO
SMS
Acrolein
β–elimination
3-Acetamidopropanal
PAO
H_2O_2
3-Acetamidopropanal
$H_2O + O_2$
PAO
H_2O_2
$H_2O + O_2$
N1-Acetyl-spermine
Spermine
SSAT1
Acetyl-CoA
CoA

Fig S7

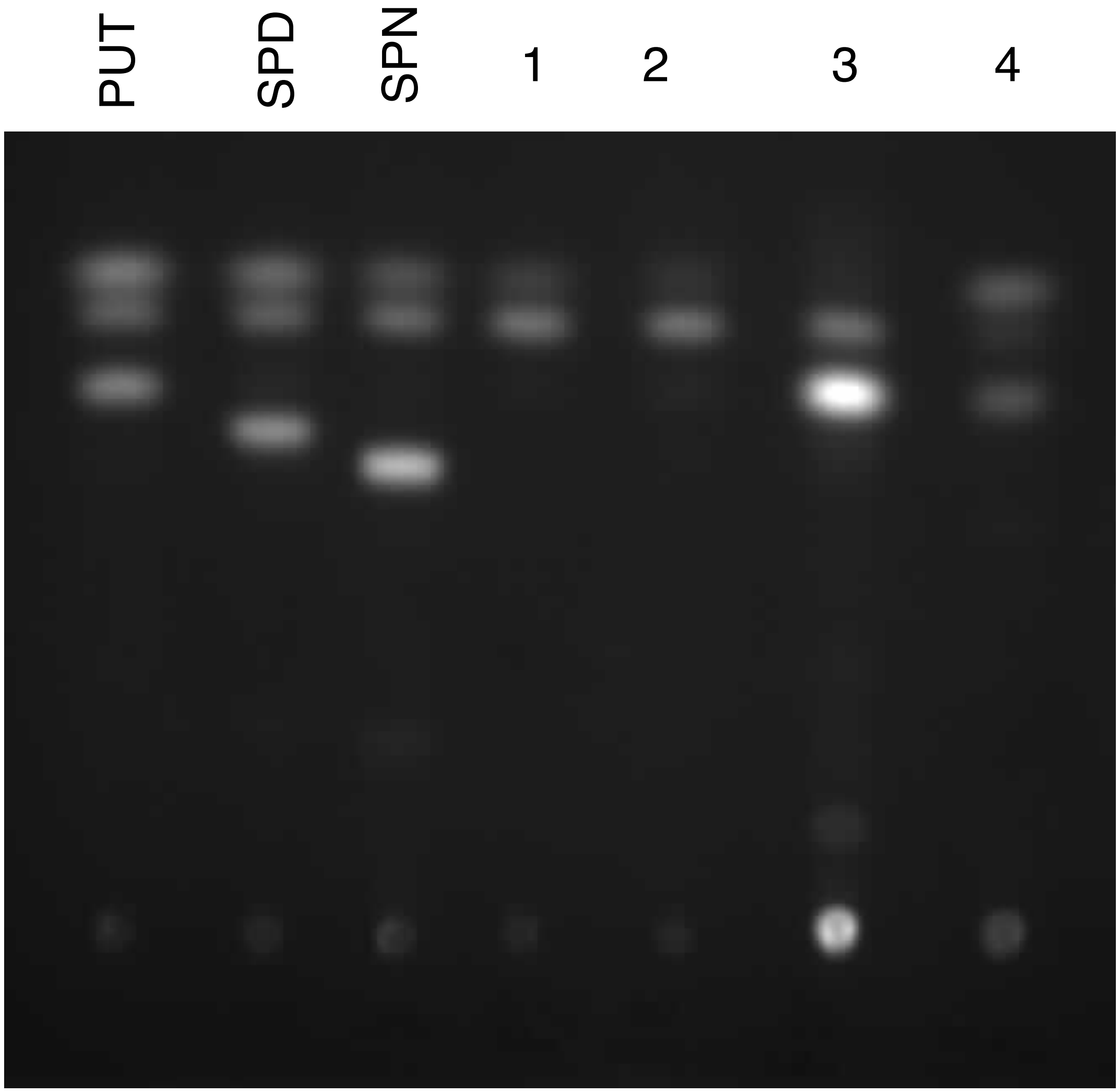

1. Mock acid (HCl) hydrolysis
2. Mock perchloric acid extraction
3. Acid (HCl) hydrolysis (stratum corneum of normal skin)
4. Perchloric acid extraction (stratum corneum of normal skin)

Fig S8

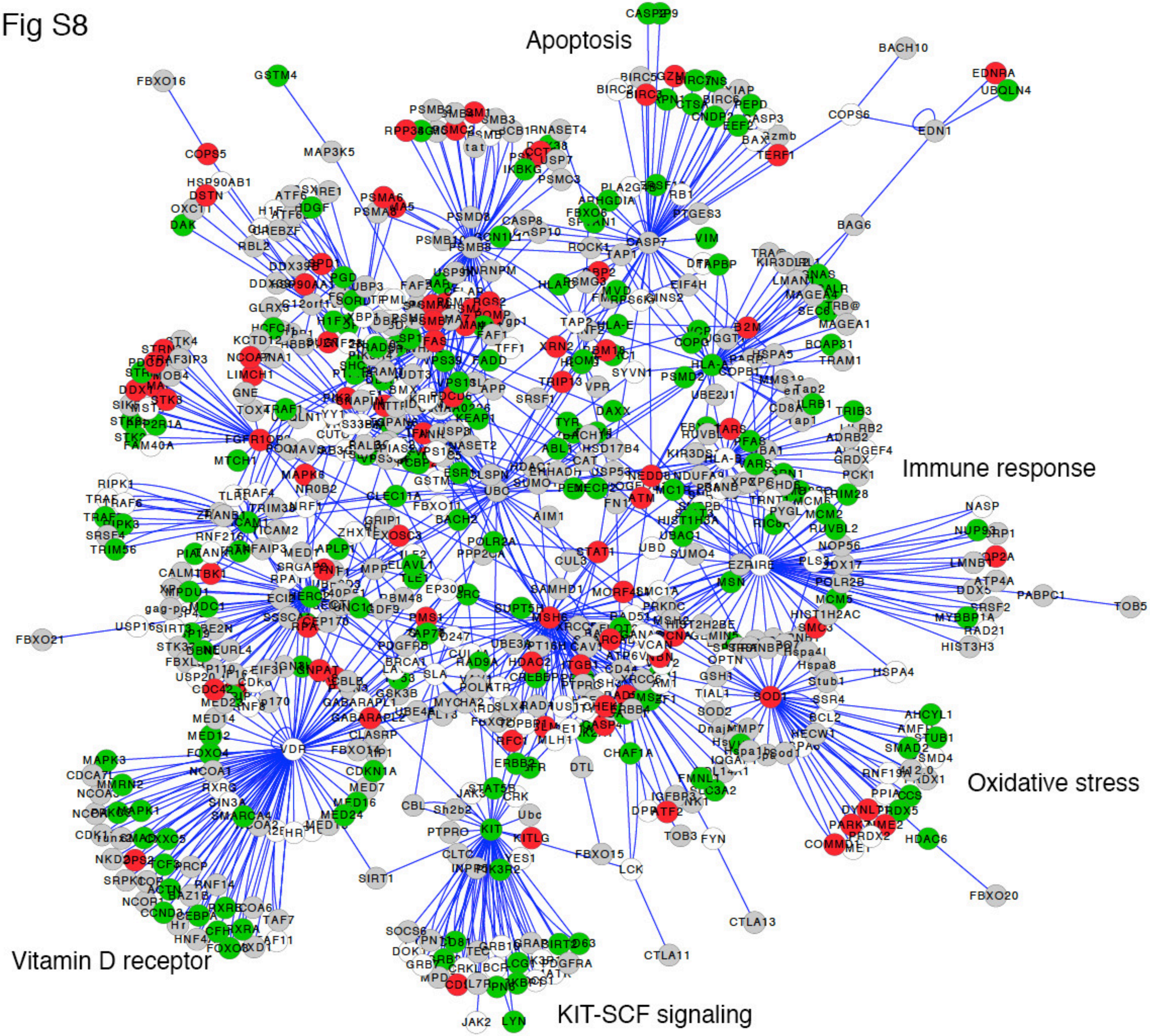
Apoptosis
Immune response
Oxidative stress
KIT-SCF signaling
Vitamin D receptor
FBXO16
GSTM4
COPS5
MAP3K5
BACH10
EDNRA
UBQLN4
COPS6
EDN1
BAG6
TERF1
CASP7
VIM
FBXO21
VDR
KIT
KITLG
LCK
FBXO15
CTLA13
CTLA11
SIRT1
SOD1
HDAC6
FBXO20
EZR
NASP
PABPC1
TOB5
JAK2
LYN

Fig S9

Vitamin D receptor

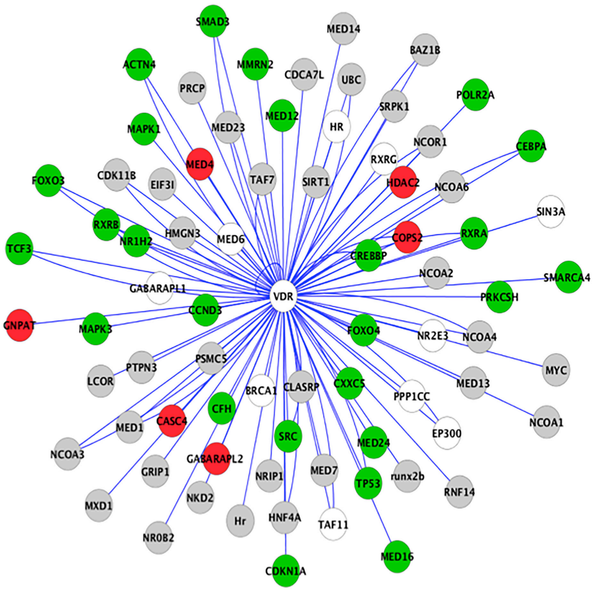

Apotosis

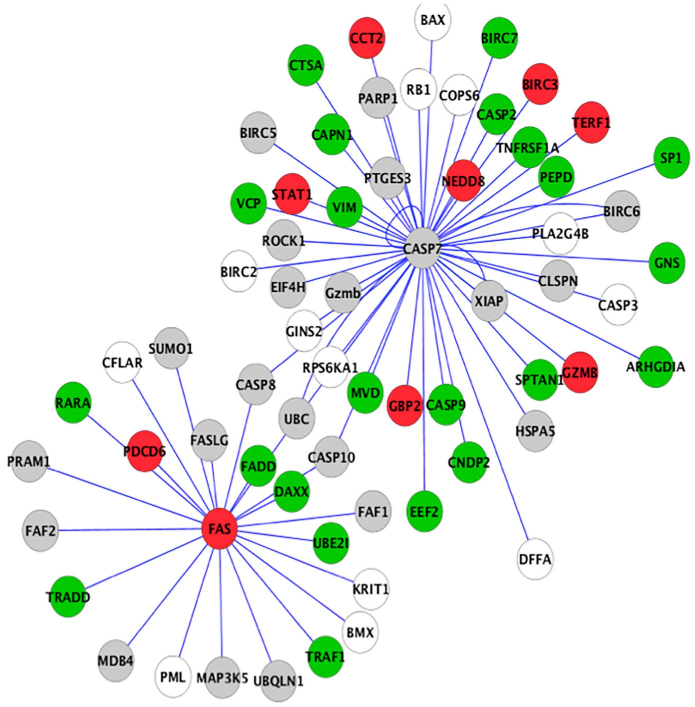

Stress response

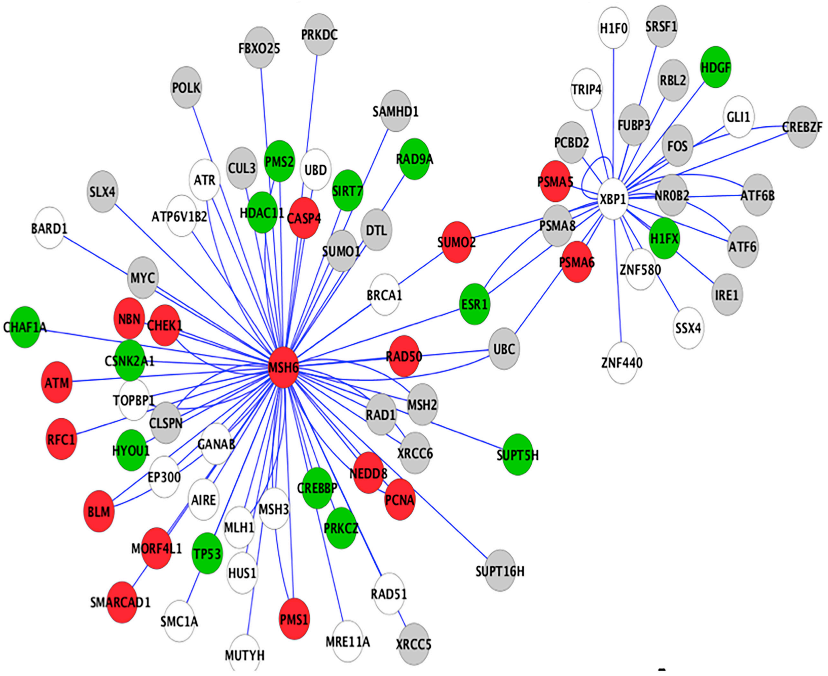

Supplementary Table 1: Vitiligo: Top 10 regulated pathways in vitiligo genome-wide transcriptional analysis

UP pathways	Genes	pVal
**RNA processing**	SR140, CWC15, WBP4, PNN, DDX46, SFRS5, RPL7, FRG1, DNAJC8, LSM3, ZCCHC6, PPWD1, RBM25, RPS24, SFRS12, SNRPA1, MRPL1, EXOSC8, EXOSC9, SF3B14, MPHOSPH10, RPL26, SSB, INTS12, CDC5L, PRPF18, CCDC76, PCF11, PPIG, UPF3B, LARP7, GTF2F2, ZRANB2, SYF2, SLU7, SNRPG, THOC1	7.47E-12
**Membrane-enclosed lumen**	ENY2, ITGB3BP, PTGS2, LYAR, MKI67IP, MBIP, SRP19, WBP4, PNN, WDR75, ATP5EP2, DDX21, TPR, TOP2B, GNL2, TOP2A, COX17, GNL3, OXR1, MRPL1, EXOSC8, EXOSC9, SSBP1, NOL8, ANAPC4, CCNL1, IFI16, CDC5L, CDK7, ZFR, GTF2B, PCF11, COQ3, MRPS18C, RPAIN, CCDC55, LARP7, ATP5C1, SLU7, SNRPG, THOC1, MRPS17, RBM34, HMGB2, SHFM1, ANAPC10, KIAA0020, ERCC5, DDX46, FRG1, GPSM2, LEO1, THBS2, RBM25, SRGN, MRPS22, MPHOSPH10, SSB, INTS12, WRN, PRPF18, SMC3, HSP90B1, PPIG, CDKN2AIP, PSMC1, GTF2F2	1.10E-10
**RNA splicing**	CWC15, WBP4, PNN, SFRS5, DDX46, FRG1, DNAJC8, LSM3, RBM25, PPWD1, SFRS12, SNRPA1, SF3B14, MPHOSPH10, CDC5L, PRPF18, PCF11, UPF3B, PPIG, GTF2F2, SYF2, ZRANB2, SLU7, THOC1, SNRPG	2.12E-10
**Ribosomal subunit**	RPL17, MRPL1, MRPS17, MRPS22, RPL26, RPL24, MRPL22, MRPS18C, RPL7, RPL6, RPL21, RPL34, RPL26L1, RPS24	1.41E-07
**Nuclear mRNA splicing, via spliceosome**	SNRPA1, PCF11, UPF3B, SFRS5, SF3B14, DNAJC8, GTF2F2, CWC15, SLU7, RBM25, SNRPG	3.88E-04

DOWN pathway	Genes	pVal
**Cornified envelope**	EVPL, SPRR2C, SPRR2A, TGM1, SPRR2B, SPRR2F, SPRR2E, CDSN	2.37E-07

**Lysosome**	PNPLA7, SGSH, C1ORF85, CLN3, GM2A, CTSD, CYBASC3, DPP7, CLCN7, CTSF, GBA	5.20E-04
**Melanin biosynthesis**	DCT, TYRP1, SILV	0.005012
**Endoplasmic reticulum membrane**	CLN3, WFS1, SILV, GPAA1, STIM1, PIGT, UBQLN4, FURIN, PNPLA6, DOLK, DHCR7, CYP26B1, DEGS2, MLANA	0.005041
**LIM domain**	ABLIM2, CRIP2, FHL3, ZYX, TRIP6, MICALL1	0.010729
**Stress fiber**	ACTN4, FHL3, ZYX, MYH14	0.013861

Supplementary table 2- List of 45 input genes for interactome study

1	IL1RN
2	DDR1
3	FAS
4	IFIH1
5	TAP2
6	AIM1
7	VDR
8	NLRP1
9	EDN1
10	CLEC11A
11	MC1R
12	TYR
13	OCA2
14	HERC2
15	KIT
16	IL10
17	HLA-A
18	MYG1
19	CCR6
20	HLA-C
21	TICAM1
22	SLA
23	MBP
24	GSTT1
25	GSTM1
26	AIRE
27	UVRAG
28	COMT
29	BACH2
30	HLA-B
31	XBP1
32	CTLA4
33	SOD1
34	FGFR1OP2
35	NRF2
36	MSH6
37	FBXO11
38	TOB2
39	CAT
40	CD44
41	PSMB9
42	RNASET2
43	PSMB8
44	CASP7
45	SOD2

Supplementary Table 3- List of protein-protein interactions for the six sub-networks (direct_pi refers to direct physical interaction between two proteins listed)

	KIT sub-network	
**Protein 1**		**Protein 2**
BCR	direct_pi	KIT
BCR	direct_pi	KIT
KIT	direct_pi	CD9
KIT	direct_pi	CD63
KIT	direct_pi	CD81
CD9	direct_pi	KIT
CD63	direct_pi	KIT
CD81	direct_pi	KIT
KIT	direct_pi	GRB2
KIT	direct_pi	PIK3R1
KIT	direct_pi	PIK3R2
KIT	direct_pi	SOCS1
SOCS1	direct_pi	KIT
TEC	direct_pi	KIT
KIT	direct_pi	CRKL
KIT	direct_pi	PIK3R1
KIT	direct_pi	PLCG1
KIT	direct_pi	INPP5D
KIT	direct_pi	YES1
GRB2	direct_pi	KIT
GRB7	direct_pi	KIT
STAT1	direct_pi	KIT
KIT	direct_pi	STAT1
STAT1	direct_pi	KIT
KIT	direct_pi	STAT1
KIT	direct_pi	CLTC
TEC	direct_pi	KIT
KIT	direct_pi	TEC
TEC	direct_pi	KIT
LCK	direct_pi	KIT
DOK1	direct_pi	KIT
LYN	direct_pi	KIT
DOK1	direct_pi	KIT
PTPN11	direct_pi	KIT
KIT	direct_pi	RFC1
KIT	direct_pi	JAK2
KIT	direct_pi	PIK3R1
KIT	direct_pi	KITLG
KIT	direct_pi	GRB10
GRB10	direct_pi	KIT

GRB10	direct_pi	KIT
PLCG1	direct_pi	KIT
MATK	direct_pi	KIT
KIT	direct_pi	PTPN6
PTPN6	direct_pi	KIT
KIT	direct_pi	PTPN6
KIT	direct_pi	PTPN11
KIT	direct_pi	MPDZ
KIT	direct_pi	MPDZ
MPDZ	direct_pi	KIT
KIT	direct_pi	MATK
LYN	direct_pi	KIT
KIT	direct_pi	LYN
CRKL	direct_pi	KIT
PTPN6	direct_pi	KIT
PTPN6	direct_pi	KIT
KIT	direct_pi	KITLG
KIT	direct_pi	PTPRO
GRAP	direct_pi	KIT
GRB2	direct_pi	KIT
SH3KBP1	direct_pi	KIT
CBLB	direct_pi	KIT
KIT	direct_pi	GRB2
KIT	direct_pi	Ubc
KIT	direct_pi	UBC
KIT	direct_pi	CBL
KIT	direct_pi	UBC
KIT	direct_pi	UBC
SOCS6	direct_pi	KIT
SOCS6	direct_pi	KIT
KIT	direct_pi	UBC
SOCS6	direct_pi	KIT
SIRT1	direct_pi	KIT
SIRT2	direct_pi	KIT
KIT	direct_pi	PIK3R1
PDGFRA	direct_pi	KIT
PIK3R1	direct_pi	KIT
KIT	direct_pi	GRB2
KIT	direct_pi	Sh2b2
KIT	direct_pi	Sh2b2
SRC	direct_pi	KIT
PTPN11	direct_pi	KIT
GRB2	direct_pi	KIT
GRB7	direct_pi	KIT

KIT	direct_pi	PIK3R1
KIT	direct_pi	CRK
CBL	direct_pi	KIT
GRB2	direct_pi	KIT
IL7R	direct_pi	KIT
KIT	direct_pi	IL7R
KIT	direct_pi	JAK3
KIT	direct_pi	STAT5B

Supplementary Table 3- List of protein-protein interactions for the six sub-networks

	VDR sub-network	
**Protein 1**		**Protein 2**
VDR	direct_pi	NCOR1
VDR	direct_pi	CREBBP
VDR	direct_pi	SRC
VDR	direct_pi	MED1
CEBPA	direct_pi	VDR
VDR	direct_pi	CEBPA
RXRA	direct_pi	VDR
VDR	direct_pi	BAZ1B
VDR	direct_pi	SMARCA4
VDR	direct_pi	TCF3
VDR	direct_pi	NCOR1
VDR	direct_pi	SIN3A
VDR	direct_pi	HDAC2
TCF3	direct_pi	VDR
BAZ1B	direct_pi	VDR
BAZ1B	direct_pi	VDR
BAZ1B	direct_pi	VDR
TCF3	direct_pi	VDR
VDR	direct_pi	SRC
VDR	direct_pi	NCOA2
VDR	direct_pi	NCOA3
LCOR	direct_pi	VDR
VDR	direct_pi	TCF3
TCF3	direct_pi	VDR
TCF3	direct_pi	VDR
TCF3	direct_pi	VDR
VDR	direct_pi	RXRG
VDR	direct_pi	SRC
VDR	direct_pi	NCOA2
VDR	direct_pi	NR2E3
VDR	direct_pi	NCOA3
VDR	direct_pi	MED1
VDR	direct_pi	POLR2A
VDR	direct_pi	RXRA
VDR	direct_pi	POLR2A
RNF14	direct_pi	VDR
NCOA4	direct_pi	VDR
VDR	direct_pi	NCOA4
VDR	direct_pi	RXRA
RXRA	direct_pi	VDR
VDR	direct_pi	RXRA

VDR	direct_pi	EP300
EP300	direct_pi	VDR
RXRA	direct_pi	VDR
VDR	direct_pi	NCOA1
RXRA	direct_pi	VDR
VDR	direct_pi	RXRA
VDR	direct_pi	NCOA1
VDR	direct_pi	VDR
COPS2	direct_pi	VDR
VDR	direct_pi	NCOR1
VDR	direct_pi	COPS2
VDR	direct_pi	TAF11
TAF11	direct_pi	VDR
MED1	direct_pi	VDR
RXRA	direct_pi	VDR
TAF7	direct_pi	VDR
VDR	direct_pi	TAF7
VDR	direct_pi	RXRA
VDR	direct_pi	NCOA1
NCOA3	direct_pi	VDR
CREBBP	direct_pi	VDR
VDR	direct_pi	SMAD3
VDR	direct_pi	RXRA
SMAD3	direct_pi	VDR
RXRA	direct_pi	VDR
SMAD3	direct_pi	VDR
RXRA	direct_pi	VDR
VDR	direct_pi	SMAD3
GRIP1	direct_pi	VDR
RXRA	direct_pi	VDR
VDR	direct_pi	MED1
VDR	direct_pi	RXRA
VDR	direct_pi	NCOA1
BAZ1B	direct_pi	VDR
TCF3	direct_pi	VDR
VDR	direct_pi	SRC
VDR	direct_pi	HNF4A
HNF4A	direct_pi	VDR
MAPK3	direct_pi	VDR
MAPK1	direct_pi	VDR
RXRA	direct_pi	VDR
runx2b	direct_pi	VDR
VDR	direct_pi	TP53
TP53	direct_pi	VDR

VDR	direct_pi	HR
VDR	direct_pi	FOXO3
VDR	direct_pi	FOXO4
VDR	direct_pi	NCOA3
VDR	direct_pi	SIRT1
VDR	direct_pi	PPP1CC
FOXO3	direct_pi	VDR
VDR	direct_pi	FOXO3
VDR	direct_pi	SIRT1
VDR	direct_pi	MED12
VDR	direct_pi	MED13
VDR	direct_pi	MED1
VDR	direct_pi	MED14
VDR	direct_pi	MED24
VDR	direct_pi	MED23
VDR	direct_pi	MED16
VDR	direct_pi	MED4
VDR	direct_pi	MED7
VDR	direct_pi	MED6
VDR	direct_pi	MED12
VDR	direct_pi	MED1
VDR	direct_pi	MED14
VDR	direct_pi	CDKN1A
VDR	direct_pi	ACTN4
ACTN4	direct_pi	VDR
VDR	direct_pi	RXRA
VDR	direct_pi	EIF3I
VDR	direct_pi	SRC
VDR	direct_pi	NRIP1
VDR	direct_pi	HMGN3
VDR	direct_pi	RXRG
VDR	direct_pi	CLASRP
VDR	direct_pi	NR1H2
VDR	direct_pi	GABARAPL2
VDR	direct_pi	GNPAT
VDR	direct_pi	NR0B2
VDR	direct_pi	RXRB
VDR	direct_pi	GABARAPL1
VDR	direct_pi	CASC4
SRC	direct_pi	VDR
MED1	direct_pi	VDR
VDR	direct_pi	SRC
VDR	direct_pi	MED1
VDR	direct_pi	CDK11B

CDK11B	direct_pi	VDR
VDR	direct_pi	UBC
VDR	direct_pi	PPP1CC
VDR	direct_pi	PPP1CC
VDR	direct_pi	PPP1CC
NCOA6	direct_pi	VDR
NCOA6	direct_pi	VDR
UBC	direct_pi	VDR
VDR	direct_pi	SRC
NCOR1	direct_pi	VDR
RXRA	direct_pi	VDR
VDR	direct_pi	NCOR1
VDR	direct_pi	NCOA1
VDR	direct_pi	PSMC5
VDR	direct_pi	UBC
VDR	direct_pi	BRCA1
VDR	direct_pi	RXRA
MYC	direct_pi	VDR
MXD1	direct_pi	VDR
Hr	direct_pi	VDR
RXRA	direct_pi	VDR
VDR	direct_pi	HR
VDR	direct_pi	HR
UBC	direct_pi	VDR
VDR	direct_pi	PTPN3
VDR	direct_pi	RXRA
VDR	direct_pi	RXRA
VDR	direct_pi	RXRA
CCND3	direct_pi	VDR
CFH	direct_pi	VDR
CXXC5	direct_pi	VDR
RXRB	direct_pi	VDR
PRCP	direct_pi	VDR
PRKCSH	direct_pi	VDR
RXRA	direct_pi	VDR
CDCA7L	direct_pi	VDR
MMRN2	direct_pi	VDR
NKD2	direct_pi	VDR
SRPK1	direct_pi	VDR

Supplementary Table 3- List of protein-protein interactions for the six sub-networks

	AIRE-HLA-PSMB sub-network	
**Protein 1**		**Protein 2**
UBC	direct_pi	PSMB8
tat	direct_pi	PSMB8
PSMA4	direct_pi	PSMB8
PSMA2	direct_pi	PSMB8
PSMB1	direct_pi	PSMB8
PSMB9	direct_pi	PSMB8
PSMB8	direct_pi	POMP
POMP	direct_pi	PSMB8
PSMB8	direct_pi	POMP
PSMA1	direct_pi	PSMB8
PSMA2	direct_pi	PSMB8
USP7	direct_pi	PSMB8
PSMB8	direct_pi	ABCB1
PSMB8	direct_pi	TAP1
PSMB8	direct_pi	TAP2
ABCB1	direct_pi	PSMB8
TAP1	direct_pi	PSMB8
PSMB1	direct_pi	PSMB8
PSMB4	direct_pi	PSMB8
PSMB8	direct_pi	PSMB3
PSMB8	direct_pi	PSMB1
PSMB8	direct_pi	PSMB6
PSMB8	direct_pi	PSMB7
PSMB8	direct_pi	PSMB8
PSMB8	direct_pi	PSMB9
PSMB8	direct_pi	PSMB10
PSMB8	direct_pi	POMP
POMP	direct_pi	PSMB8
PSMA6	direct_pi	PSMB8
PSMB7	direct_pi	PSMB8
PSMB8	direct_pi	HCVgp1
HCVgp1	direct_pi	PSMB8
PSMA4	direct_pi	PSMB8
PSMB6	direct_pi	PSMB8
PSME1	direct_pi	PSMB8
PSME2	direct_pi	PSMB8
UBC	direct_pi	PSMB8
UBC	direct_pi	PSMB8
PSMB7	direct_pi	PSMB8
PSMA2	direct_pi	PSMB8
PSMB4	direct_pi	PSMB8

PSMA5	direct_pi	PSMB8
PSMB5	direct_pi	PSMB8
PSMA3	direct_pi	PSMB8
PSMA1	direct_pi	PSMB8
PSMB2	direct_pi	PSMB8
PSMB6	direct_pi	PSMB8
PSMB1	direct_pi	PSMB8
PSMA6	direct_pi	PSMB8
PSMA4	direct_pi	PSMB8
PSMB3	direct_pi	PSMB8
PSMA7	direct_pi	PSMB8
PSMA8	direct_pi	PSMB8
PSMC5	direct_pi	PSMB8
PSMC1	direct_pi	PSMB8
PSMC2	direct_pi	PSMB8
PSMB8	direct_pi	PSMD8
PSMC4	direct_pi	PSMB8
PSMC3	direct_pi	PSMB8
PSMB8	direct_pi	USP9X
PSMA2	direct_pi	PSMB8
LSM1	direct_pi	PSMB8
PSMB8	direct_pi	GCN1L1
PSMB8	direct_pi	HNRNPM
PSMB8	direct_pi	PSMA2
PSMB8	direct_pi	PSMA3
PSMB8	direct_pi	PSMA4
PSMB8	direct_pi	PSMA5
PSMB8	direct_pi	PSMB1
PSMB8	direct_pi	PSMB4
PSMB8	direct_pi	PSMB5
PSMB8	direct_pi	PSMB6
PSMB8	direct_pi	PSMB7
CCT2	direct_pi	PSMB8
DHX38	direct_pi	PSMB8
EIF4G1	direct_pi	PSMB8
IKBKG	direct_pi	PSMB8
PSMA1	direct_pi	PSMB8
PSMA6	direct_pi	PSMB8
PSMB2	direct_pi	PSMB8
RPP38	direct_pi	PSMB8
UBC	direct_pi	PSMB8
tat	direct_pi	PSMB8
PSMA4	direct_pi	PSMB8
PSMA2	direct_pi	PSMB8

PSMB1	direct_pi	PSMB8
PSMB9	direct_pi	PSMB8
PSMB8	direct_pi	POMP
POMP	direct_pi	PSMB8
PSMB8	direct_pi	POMP
PSMA1	direct_pi	PSMB8
PSMA2	direct_pi	PSMB8
USP7	direct_pi	PSMB8
PSMB8	direct_pi	ABCB1
PSMB8	direct_pi	TAP1
PSMB8	direct_pi	TAP2
ABCB1	direct_pi	PSMB8
TAP1	direct_pi	PSMB8
PSMB1	direct_pi	PSMB8
PSMB4	direct_pi	PSMB8
PSMB8	direct_pi	PSMB3
PSMB8	direct_pi	PSMB1
PSMB8	direct_pi	PSMB6
PSMB8	direct_pi	PSMB7
PSMB8	direct_pi	PSMB8
PSMB8	direct_pi	PSMB9
PSMB8	direct_pi	PSMB10
PSMB8	direct_pi	POMP
POMP	direct_pi	PSMB8
PSMA6	direct_pi	PSMB8
PSMB7	direct_pi	PSMB8
PSMB8	direct_pi	HCVgp1
HCVgp1	direct_pi	PSMB8
PSMA4	direct_pi	PSMB8
PSMB6	direct_pi	PSMB8
PSME1	direct_pi	PSMB8
PSME2	direct_pi	PSMB8
UBC	direct_pi	PSMB8
UBC	direct_pi	PSMB8
PSMB7	direct_pi	PSMB8
PSMA2	direct_pi	PSMB8
PSMB4	direct_pi	PSMB8
PSMA5	direct_pi	PSMB8
PSMB5	direct_pi	PSMB8
PSMA3	direct_pi	PSMB8
PSMA1	direct_pi	PSMB8
PSMB2	direct_pi	PSMB8
PSMB6	direct_pi	PSMB8
PSMB1	direct_pi	PSMB8

PSMA6	direct_pi	PSMB8
PSMA4	direct_pi	PSMB8
PSMB3	direct_pi	PSMB8
PSMA7	direct_pi	PSMB8
PSMA8	direct_pi	PSMB8
PSMC5	direct_pi	PSMB8
PSMC1	direct_pi	PSMB8
PSMC2	direct_pi	PSMB8
PSMB8	direct_pi	PSMD8
PSMC4	direct_pi	PSMB8
PSMC3	direct_pi	PSMB8
PSMB8	direct_pi	USP9X
PSMA2	direct_pi	PSMB8
LSM1	direct_pi	PSMB8
PSMB8	direct_pi	GCN1L1
PSMB8	direct_pi	HNRNPM
PSMB8	direct_pi	PSMA2
PSMB8	direct_pi	PSMA3
PSMB8	direct_pi	PSMA4
PSMB8	direct_pi	PSMA5
PSMB8	direct_pi	PSMB1
PSMB8	direct_pi	PSMB4
PSMB8	direct_pi	PSMB5
PSMB8	direct_pi	PSMB6
PSMB8	direct_pi	PSMB7
CCT2	direct_pi	PSMB8
DHX38	direct_pi	PSMB8
EIF4G1	direct_pi	PSMB8
IKBKG	direct_pi	PSMB8
PSMA1	direct_pi	PSMB8
PSMA6	direct_pi	PSMB8
PSMB2	direct_pi	PSMB8
RPP38	direct_pi	PSMB8
CREBBP	direct_pi	AIRE
AIRE	direct_pi	AIRE
AIRE	direct_pi	AIRE
CREBBP	direct_pi	AIRE
AIRE	direct_pi	AIRE
AIRE	direct_pi	AIRE
CREBBP	direct_pi	AIRE
CREBBP	direct_pi	AIRE
AIRE	direct_pi	MYBBP1A
AIRE	direct_pi	NOP56
AIRE	direct_pi	DDX5

AIRE	direct_pi	DDX17
AIRE	direct_pi	RUVBL2
AIRE	direct_pi	TOP2A
AIRE	direct_pi	PRKDC
AIRE	direct_pi	SSRP1
AIRE	direct_pi	PARP1
AIRE	direct_pi	POLR2A
AIRE	direct_pi	PCNA
AIRE	direct_pi	POLR2B
AIRE	direct_pi	MCM2
AIRE	direct_pi	MCM5
AIRE	direct_pi	MCM6
AIRE	direct_pi	MSH6
AIRE	direct_pi	HIST1H2AC
AIRE	direct_pi	HIST1H2BO
AIRE	direct_pi	MSH2
AIRE	direct_pi	NASP
AIRE	direct_pi	RAD21
AIRE	direct_pi	SMC1A
AIRE	direct_pi	SMC3
AIRE	direct_pi	CHD6
AIRE	direct_pi	TRIM28
AIRE	direct_pi	LMNB1
AIRE	direct_pi	KPNB1
AIRE	direct_pi	XPO1
AIRE	direct_pi	IPO7
AIRE	direct_pi	XPOT
AIRE	direct_pi	RANBP2
AIRE	direct_pi	RANBP9
AIRE	direct_pi	NUP93
AIRE	direct_pi	SRSF3
AIRE	direct_pi	SRSF1
AIRE	direct_pi	PABPC1
AIRE	direct_pi	SRSF2
AIRE	direct_pi	SNRPD3
AIRE	direct_pi	EFTUD2
AIRE	direct_pi	SNRPB
AIRE	direct_pi	GEMIN5
PRKDC	direct_pi	AIRE
SSRP1	direct_pi	AIRE
TOP2A	direct_pi	AIRE
LMNB1	direct_pi	AIRE
NUP93	direct_pi	AIRE
NASP	direct_pi	AIRE

DDX5	direct_pi	AIRE
SRSF2	direct_pi	AIRE
MYBBP1A	direct_pi	AIRE
TRIM28	direct_pi	AIRE
RAD21	direct_pi	AIRE
SMC1A	direct_pi	AIRE
AIRE	direct_pi	HSPA5
AIRE	direct_pi	HSPA4
AIRE	direct_pi	HIST1H3A
AIRE	direct_pi	HIST2H2BE
AIRE	direct_pi	HIST1H3A
AIRE	direct_pi	HIST1H3A
AIRE	direct_pi	HIST1H3A
AIRE	direct_pi	UBAC1
AIRE	direct_pi	DAXX
AIRE	direct_pi	DAXX
AIRE	direct_pi	AIRE
AIRE	direct_pi	HDAC1
AIRE	direct_pi	HDAC2
HIST3H3	direct_pi	AIRE
ATP4A	direct_pi	AIRE
HLA-B	direct_pi	PFAS
HLA-B	direct_pi	VCP
HLA-B	direct_pi	AHCY
HLA-B	direct_pi	VARS
HLA-B	direct_pi	EZR
HLA-B	direct_pi	TARS
HLA-B	direct_pi	PLS3
HLA-B	direct_pi	PAICS
HLA-B	direct_pi	RUVBL1
HLA-B	direct_pi	PSMD1
HLA-B	direct_pi	TRIM28
HLA-B	direct_pi	MSN
LILRB2	direct_pi	HLA-B
LILRB1	direct_pi	HLA-B
HLA-B	direct_pi	CD8A
HLA-B	direct_pi	KIR3DS1
TRIB3	direct_pi	HLA-B
ARHGEF4	direct_pi	HLA-B
PCK1	direct_pi	HLA-B
UBC	direct_pi	HLA-B
UBC	direct_pi	HLA-B
Tap2	direct_pi	HLA-B
HLA-B	direct_pi	B2M

Tap1	direct_pi	HLA-B
Tap2	direct_pi	HLA-B
Tap1	direct_pi	HLA-B
UBC	direct_pi	HLA-B
UBD	direct_pi	HLA-B
HLA-B	direct_pi	NDUFA9
GRDX	direct_pi	HLA-B
env	direct_pi	HLA-B
MMS19	direct_pi	HLA-B
ADRB2	direct_pi	HLA-B
HLA-B	direct_pi	STAT1
HLA-B	direct_pi	HSPA5
UBC	direct_pi	HLA-B
NEDD8	direct_pi	HLA-B
SUMO1	direct_pi	HLA-B
MAGEA4	direct_pi	HLA-A
MAGEA1	direct_pi	HLA-A
KIR3DL2	direct_pi	HLA-A
TRA@	direct_pi	HLA-A
TRB@	direct_pi	HLA-A
TAPBP	direct_pi	HLA-A
TAP1	direct_pi	HLA-A
HLA-A	direct_pi	COPB1
HLA-A	direct_pi	COPG
HLA-A	direct_pi	TAPBP
HLA-A	direct_pi	TAP1
HLA-A	direct_pi	UBC
UBC	direct_pi	HLA-A
HLA-A	direct_pi	SYVN1
HLA-A	direct_pi	UBC
HLA-A	direct_pi	UBE2J1
HLA-A	direct_pi	DERL1
HLA-A	direct_pi	PSMD2
HLA-A	direct_pi	UBC
UBC	direct_pi	HLA-A
UBC	direct_pi	HLA-A
UBC	direct_pi	HLA-A
UBC	direct_pi	HLA-A
TAP1	direct_pi	HLA-A
TRAM1	direct_pi	HLA-A
HLA-A	direct_pi	UBC
SEC61A1	direct_pi	HLA-A
Tap1	direct_pi	HLA-A
Tap2	direct_pi	HLA-A

BCAP31	direct_pi	HLA-A
LMAN1	direct_pi	HLA-A
HLA-A	direct_pi	MAGEA4
HLA-A	direct_pi	BCAP31
UBC	direct_pi	HLA-A
HLA-A	direct_pi	B2M
HLA-A	direct_pi	B2M
TAP2	direct_pi	HLA-A
env	direct_pi	HLA-A
UBC	direct_pi	HLA-A
UBC	direct_pi	HLA-A
MMS19	direct_pi	HLA-A
CALR	direct_pi	HLA-A
ATM	direct_pi	HLA-A
HLA-A	direct_pi	STAT3
GNAS	direct_pi	HLA-A
HLA-A	direct_pi	GNAS
HLA-A	direct_pi	UBC
HLA-A	direct_pi	BAG6
BAG6	direct_pi	HLA-A
VCP	direct_pi	HLA-A
UBC	direct_pi	HLA-A
NEDD8	direct_pi	HLA-A
SUMO1	direct_pi	HLA-A

Supplementary Table 3- List of protein-protein interactions for the six sub-networks

XBP1-MSH6 sub-network

**Protein 1**		**Protein 2**
ESR1	direct_pi	XBP1
XBP1	direct_pi	ESR1
XBP1	direct_pi	FOS
IRE1	direct_pi	XBP1
GLI1	direct_pi	XBP1
H1F0	direct_pi	XBP1
HDGF	direct_pi	XBP1
RBL2	direct_pi	XBP1
SRSF1	direct_pi	XBP1
SSX4	direct_pi	XBP1
XBP1	direct_pi	NR0B2
XBP1	direct_pi	FUBP3
XBP1	direct_pi	H1FX
XBP1	direct_pi	TRIP4
XBP1	direct_pi	ZNF580
XBP1	direct_pi	PCBD2
XBP1	direct_pi	ZNF440
XBP1	direct_pi	PSMA5
XBP1	direct_pi	PSMA6
XBP1	direct_pi	PSMA8
SUMO2	direct_pi	XBP1
UBC	direct_pi	XBP1
SUMO2	direct_pi	XBP1
UBC	direct_pi	XBP1
UBC	direct_pi	XBP1
ATF6B	direct_pi	XBP1
ATF6	direct_pi	XBP1
XBP1	direct_pi	ATF6B
XBP1	direct_pi	ATF6
XBP1	direct_pi	XBP1
XBP1	direct_pi	CREBZF
CREBZF	direct_pi	XBP1
BRCA1	direct_pi	MSH6
CASP4	direct_pi	MSH6
MYC	direct_pi	MSH6
BRCA1	direct_pi	MSH6
MSH6	direct_pi	BLM
MSH6	direct_pi	ATM
MSH6	direct_pi	MLH1
BARD1	direct_pi	MSH6
BRCA1	direct_pi	MSH6

MRE11A	direct_pi	MSH6
MSH2	direct_pi	MSH6
NBN	direct_pi	MSH6
RAD50	direct_pi	MSH6
MSH6	direct_pi	RFC1
MSH2	direct_pi	MSH6
MSH6	direct_pi	SMC1A
MSH2	direct_pi	MSH6
MSH6	direct_pi	PCNA
MSH6	direct_pi	PCNA
MSH6	direct_pi	PCNA
PCNA	direct_pi	MSH6
MSH6	direct_pi	MSH2
MSH2	direct_pi	MSH6
MSH2	direct_pi	MSH6
PMS2	direct_pi	MSH6
AIRE	direct_pi	MSH6
MSH6	direct_pi	ATR
MSH6	direct_pi	TOPBP1
MSH6	direct_pi	CLSPN
MSH6	direct_pi	CHEK1
ATR	direct_pi	MSH6
CLSPN	direct_pi	MSH6
TOPBP1	direct_pi	MSH6
CHEK1	direct_pi	MSH6
UBC	direct_pi	MSH6
MSH6	direct_pi	UBC
PRKCZ	direct_pi	MSH6
BRCA1	direct_pi	MSH6
ESR1	direct_pi	MSH6
MORF4L1	direct_pi	MSH6
MYC	direct_pi	MSH6
EP300	direct_pi	MSH6
CREBBP	direct_pi	MSH6
BARD1	direct_pi	MSH6
SUMO1	direct_pi	MSH6
UBC	direct_pi	MSH6
UBC	direct_pi	MSH6
PCNA	direct_pi	MSH6
DTL	direct_pi	MSH6
MSH6	direct_pi	CHAF1A
MSH6	direct_pi	CHAF1A
MSH6	direct_pi	CHAF1A
MSH6	direct_pi	PCNA

SMARCAD1	direct_pi	MSH6
UBC	direct_pi	MSH6
UBC	direct_pi	MSH6
UBC	direct_pi	MSH6
MSH6	direct_pi	MSH2
MSH6	direct_pi	MLH1
MSH6	direct_pi	PMS2
MSH6	direct_pi	PMS1
MLH1	direct_pi	MSH6
MSH2	direct_pi	MSH6
PMS2	direct_pi	MSH6
PMS1	direct_pi	MSH6
SUMO2	direct_pi	MSH6
SUMO2	direct_pi	MSH6
BLM	direct_pi	MSH6
TP53	direct_pi	MSH6
RAD51	direct_pi	MSH6
MSH6	direct_pi	BLM
MSH6	direct_pi	MSH2
XRCC6	direct_pi	MSH6
XRCC6	direct_pi	MSH6
MSH6	direct_pi	XRCC6
MSH6	direct_pi	XRCC5
MSH6	direct_pi	PRKDC
SIRT7	direct_pi	MSH6
FBXO25	direct_pi	MSH6
NEDD8	direct_pi	MSH6
CUL3	direct_pi	MSH6
SLX4	direct_pi	MSH6
HUS1	direct_pi	MSH6
RAD1	direct_pi	MSH6
RAD9A	direct_pi	MSH6
HUS1	direct_pi	MSH6
RAD1	direct_pi	MSH6
RAD9A	direct_pi	MSH6
MUTYH	direct_pi	MSH6
MUTYH	direct_pi	MSH6
UBC	direct_pi	MSH6
UBC	direct_pi	MSH6
MSH6	direct_pi	MLH1
MSH6	direct_pi	PMS2
MLH1	direct_pi	MSH6
MSH2	direct_pi	MSH6
MSH6	direct_pi	PCNA

MSH3	direct_pi	MSH6
MSH6	direct_pi	SUPT16H
MSH2	direct_pi	MSH6
CSNK2A1	direct_pi	MSH6
UBC	direct_pi	MSH6
UBC	direct_pi	MSH6
MSH2	direct_pi	MSH6
MSH6	direct_pi	PCNA
HDAC11	direct_pi	MSH6
UBD	direct_pi	MSH6
ATP6V1B2	direct_pi	MSH6
GANAB	direct_pi	MSH6
HYOU1	direct_pi	MSH6
XRCC6	direct_pi	MSH6
MSH6	direct_pi	SAMHD1
MSH6	direct_pi	SUPT5H
POLK	direct_pi	MSH6

Supplementary Table 3- List of protein-protein interactions for the six sub-networks

FAS-CASP sub-network

**Protein 1**		**Protein 2**
FAS	direct_pi	FADD
FAS	direct_pi	DAXX
FAS	direct_pi	FASLG
FAS	direct_pi	FAF1
FAS	direct_pi	PDCD6
FAS	direct_pi	UBE2I
FAS	direct_pi	SUMO1
FAS	direct_pi	CASP8
FAS	direct_pi	CASP10
FAS	direct_pi	UBC
FAS	direct_pi	KRIT1
FAS	direct_pi	CFLAR
FAS	direct_pi	BMX
FAS	direct_pi	RARA
FAS	direct_pi	TRAF1
FAS	direct_pi	PRAM1
FAS	direct_pi	UBQLN1
FAS	direct_pi	FAF2
FAS	direct_pi	MAP3K5
FAS	direct_pi	TRADD
FAS	direct_pi	PML
FAS	direct_pi	MDB4
FAS	direct_pi	UBE2I
HSPA5	direct_pi	CASP7
CASP7	direct_pi	CASP2
BIRC7	direct_pi	CASP7
BIRC5	direct_pi	CASP7
XIAP	direct_pi	CASP7
CASP8	direct_pi	CASP7
BIRC3	direct_pi	CASP7
BIRC2	direct_pi	CASP7
XIAP	direct_pi	CASP7
CASP8	direct_pi	CASP7
CASP10	direct_pi	CASP7
CASP7	direct_pi	TNFRSF1A
BIRC5	direct_pi	CASP7
BIRC5	direct_pi	CASP7
CASP7	direct_pi	XIAP
XIAP	direct_pi	CASP7
XIAP	direct_pi	CASP7
BIRC2	direct_pi	CASP7

CASP7	direct_pi	UBC
BIRC2	direct_pi	CASP7
UBC	direct_pi	CASP7
BIRC6	direct_pi	CASP7
XIAP	direct_pi	CASP7
TERF1	direct_pi	CASP7
NEDD8	direct_pi	CASP7
CASP7	direct_pi	UBC
CASP7	direct_pi	COPS6
CASP7	direct_pi	BIRC6
BIRC2	direct_pi	CASP7
XIAP	direct_pi	CASP7
XIAP	direct_pi	CASP7
BIRC2	direct_pi	CASP7
BIRC3	direct_pi	CASP7
CASP7	direct_pi	XIAP
XIAP	direct_pi	CASP7
CASP7	direct_pi	XIAP
XIAP	direct_pi	CASP7
CASP7	direct_pi	XIAP
XIAP	direct_pi	CASP7
XIAP	direct_pi	CASP7
CASP7	direct_pi	XIAP
BAX	direct_pi	CASP7
CASP7	direct_pi	SP1
CCT2	direct_pi	CASP7
CCT2	direct_pi	CASP7
VCP	direct_pi	CASP7
BIRC2	direct_pi	CASP7
UBC	direct_pi	CASP7
CASP7	direct_pi	RB1
CASP7	direct_pi	RB1
CASP7	direct_pi	PLA2G4B
CASP7	direct_pi	PARP1
CASP7	direct_pi	CLSPN
CASP7	direct_pi	PTGES3
CASP7	direct_pi	ARHGDIA
CASP7	direct_pi	PARP1
CASP7	direct_pi	VIM
CASP7	direct_pi	SPTAN1
CASP7	direct_pi	STAT1
CASP7	direct_pi	DFFA
CASP7	direct_pi	ROCK1
CASP3	direct_pi	CASP7

CASP7	direct_pi	CASP7
CASP7	direct_pi	CASP9
GZMB	direct_pi	CASP7
Gzmb	direct_pi	CASP7
CAPN1	direct_pi	CASP7
CNDP2	direct_pi	CASP7
EEF2	direct_pi	CASP7
GNS	direct_pi	CASP7
PEPD	direct_pi	CASP7
CTSA	direct_pi	CASP7
CASP7	direct_pi	EIF4H
CASP7	direct_pi	GBP2
CASP7	direct_pi	GINS2
CASP7	direct_pi	MVD
CASP7	direct_pi	RPS6KA1

Supplementary Table 3- List of protein-protein interactions for the six sub-networks

	SOD-CAT sub-network	
**Protein 1**		**Protein 2**
BCL2	direct_pi	SOD1
SOD1	direct_pi	BCL2
BCL2	direct_pi	SOD1
SOD1	direct_pi	BCL2
SOD1	direct_pi	HECW1
SOD1	direct_pi	SSR4
CCS	direct_pi	SOD1
CCS	direct_pi	SOD1
RNF19A	direct_pi	SOD1
SOD1	direct_pi	UBC
PARK7	direct_pi	SOD1
SOD1	direct_pi	HSPA8
SOD1	direct_pi	HSPA4
PSMD4	direct_pi	SOD1
SOD1	direct_pi	STUB1
SOD1	direct_pi	HSPA4
SOD1	direct_pi	UBC
HSPA4	direct_pi	SOD1
RNF19A	direct_pi	SOD1
SOD1	direct_pi	UBC
RNF19A	direct_pi	SOD1
SOD1	direct_pi	UBC
RNF19A	direct_pi	SOD1
STUB1	direct_pi	SOD1
SOD1	direct_pi	UBC
SOD1	direct_pi	CCS
SOD1	direct_pi	UBC
SOD1	direct_pi	UBC
SOD1	direct_pi	UBC
SOD1	direct_pi	UBC
AMFR	direct_pi	SOD1
AMFR	direct_pi	SOD1
AMFR	direct_pi	SOD1
DYNLT1	direct_pi	SOD1
DYNLT1	direct_pi	SOD1
SOD1	direct_pi	UBC
SOD1	direct_pi	HSPA4
UBC	direct_pi	SOD1
UBC	direct_pi	SOD1
UBC	direct_pi	SOD1
SUMO4	direct_pi	SOD1

SOD1	direct_pi	Stub1
SOD1	direct_pi	UBC
COMMD1	direct_pi	SOD1
SOD1	direct_pi	COMMD1
CCS	direct_pi	SOD1
UBE3A	direct_pi	SOD1
SOD1	direct_pi	UBC
SOD1	direct_pi	Sod1
SOD1	direct_pi	Hspa8
SOD1	direct_pi	Hspa2
SOD1	direct_pi	Hspa4l
SOD1	direct_pi	Hspa1b
SOD1	direct_pi	Hsph1
SOD1	direct_pi	Hspa4
SOD1	direct_pi	Hspa5
SOD1	direct_pi	Dnaja1
SOD1	direct_pi	Ccs
SOD1	direct_pi	Hspa8
PRDX5	direct_pi	SOD1
AHCYL1	direct_pi	SOD1
PRDX1	direct_pi	SOD1
NME2	direct_pi	SOD1
PLS3	direct_pi	SOD1
SOD1	direct_pi	SOD2
NME1	direct_pi	SOD1
SOD1	direct_pi	SPTBN1
SOD1	direct_pi	TIAL1
PRDX2	direct_pi	SOD1
PPP2CA	direct_pi	SOD1
PPIA	direct_pi	SOD1
SOD1	direct_pi	OPTN
UBC	direct_pi	SOD1
UBC	direct_pi	SOD1
SOD1	direct_pi	GSH1
SOD1	direct_pi	HDAC6
SOD1	direct_pi	UBC
SMAD2	direct_pi	SOD1
CAT	direct_pi	EHHADH
CAT	direct_pi	ABL1
CAT	direct_pi	PEX5
CAT	direct_pi	ABL2
CAT	direct_pi	CAT
CAT	direct_pi	MECP2
CAT	direct_pi	KEAP1

CAT	direct_pi	STIM1
CAT	direct_pi	SUMO4
CAT	direct_pi	USP53
CAT	direct_pi	APP
CAT	direct_pi	HSD17B4
